# Research and developmental strategies to hasten the improvement of orphan crops

**DOI:** 10.1080/21645698.2024.2423987

**Published:** 2024-12-24

**Authors:** Ufuoma Akpojotor, Olubusayo Oluwole, Olaniyi Oyatomi, Rajneesh Paliwal, Michael Abberton

**Affiliations:** a Genetic Resources Center, International Institute of Tropical Agriculture, Ibadan, Oyo state, Nigeria; b Department of Crop Production and Protection, Obafemi Awolowo University, Ile-Ife, Osun state, Nigeria

**Keywords:** Climate resilient, food and nutritional security, genebanks, high-throughput, orphan crops

## Abstract

To feed the world’s expanding population, crop breeders need to increase agricultural productivity and expand major crops base. Orphan crops are indigenously important crops with great potential because they are climate resilient, highly nutritious, contain nutraceutical compounds, and can improve the livelihood of smallholder farmers and consumers, but they have received little or no scientific attention. This review article examines several research and developmental strategies for hastening the improvement of these crops so that they can effectively play their role in securing food and nutrition. The integration of both research and developmental approaches will open up modern opportunities for crop improvement. We summarized ways in which advanced tools in phenotyping and genotyping, using high-throughput processes, can be used to accelerate their improvement. Finally, we suggest roles the genebanks can play in improving orphan crops, as the utilization of plant genetic resources is important for the genetic improvement of a crop.

## Introduction

With the exponentially growing population rate and the lack of available arable land, humanity is facing enormous challenges that have resulted in food security being threatened.^1^ Due to the limited number of stable crops and their ability to withstand climate change, scientists and breeders have shifted focus to looking at alternative methods of ensuring future food security. Breeders have discovered germplasm (local landraces or crop wild relatives) with advantageous traits and have introduced the adaptive alleles into superior varieties. Despite significant efforts and numerous breakthroughs in developing climate-resilient crops, yields have begun to plateau due to the adverse effects of extreme weather conditions in some years and some regions of the world.^2^ Neglected, orphan, underutilized, or opportunity crops offer an important connection in the ongoing problem of food security. These scientifically ignored crops are specially adapted to the local environmental circumstances, are used as part of local meals, and have contributed to the economic stability of farmers.^3^ They serve as a large gene pool for advancing crops in the future and are crucial to the world’s food and nutrition security. They may also help in ensuring the sustainability of food systems in the face of climate change.^4^

Research attention is shifting toward orphan crops as a result of a renewed awareness of their potential as nutrient-dense, adaptable to a variety of food systems, and tolerant of suboptimal growing conditions.^5^ The potential orphan crops possess has made them important in ensuring food and nutritional security as well as agricultural sustainability. They are well suited for sustainable agriculture since they are more resilient to biotic and abiotic stressors and make better use of water and soil nutrients. The climate resilience traits some orphan crops possess have made them significant in ensuring the continued production of food in these climate-changing times.^6^ Due to their nutritious nature, they have the potential to improve nutritional security and their inclusion to complement major crops in diet and farming practices ensure food system and agricultural diversification. Additionally, some of them contribute to sustainable and healthy diets as they possess medicinal properties, exemplifying functional foods, thereby highlighting their value in transforming food systems.^7^ Furthermore, they can strengthen the economies of emerging nations because they require low input to cultivate therefore, are a good source of income for small and marginal farmholders.^8^

Specialized breeding initiatives are needed to identify these crops and their potential cultivars and ensure their commercial viability. Orphan crops are seldom taken into consideration in industrialized nations where breeding programs run by private businesses predominate over public ones, due to the ongoing high research interest in major crops and their widespread distribution, minor crops and their related indigenous knowledge are lost, and the development of true-breeding, high-yielding cultivars of potential significance for global food systems are slowed down by this lack of investment.^9,10^ However, if all these hurdles are overcome, the inclusion of orphan crops in our food system can help double food production by 2050, diversify and build a climate-resilient food system, and improve the diet of the populace as well as the income of small farm owners.^6,11^ To achieve continued research and development on orphan crops, the collection of both ex-situ and in-situ germplasm is vital, followed by their exhaustive characterization. Although bodies like the International Institute of Tropical Agriculture (IITA), Alliance of Bioversity International, International Center for Tropical Agriculture (CIAT), African Orphan Crops Consortium (AOCC), and some other international agencies are making significant efforts, more organizations need to intensify their efforts toward continued research and development on these crops.^5,12^ The objective of this review is to underscore research and developmental strategies as well as suggest pre-breeding programs that can be carried out in the genebanks to accelerate the improvement of orphan crops. These methods may help fast-track the improvement of orphan crops without affecting their valuable traits.

## Recent research areas, achievements, and gaps in crop improvement of some orphan crops

### Hyacinth Bean (Lablab Purpureus L. Sweet)

This is a grain legume of African origin that grows well in diverse environmental conditions and is extremely resistant to drought. As a result, it is commonly grown throughout Asia and Africa’s tropical and subtropical climates. It was confirmed that domesticated Lablab had dual origin: the widely distributed four-seeded type and the locally confined (to Ethiopia) two-seeded type are genetically distinct from one other, and domestication processes have occurred in both of these groups.^13,14^ Described an inclusive African-led project to generate a high-quality reference genome for lablab, a versatile and climate-resilient crop. In addition to highlighting some intriguing and significant aspects of the lablab genome, as well as its domestication and population diversity, the chromosome-scale assembly of Lablab improves upon the prior assembly in a number of ways.^15,16^ Identified 22 and 2567 SSR markers, some of which were used in the population genetics that clearly showed a distinction between the 2-seeded and 4-seeded pod.^17^ The use of molecular markers for the breeding of this crop is still in its early stages. The identification of single gene traits such as bruchid resistance, maturity, and photoperiod sensitivity, as well as the development of a variety that combines both abiotic and biotic stresses, should be the focus of the breeding program for Lablab. In addition, the assembly and annotation of a more complete reference genome should be targeted.^14^

### Winged Bean (Psophocarpus Tetragonolobus (L.) DC.)

Winged bean is cultivated mainly for its pods, seeds, and tubers. It is one of those crops that has benefited from selective breeding by creating lines that are less photoperiod-vulnerable.^18^ All parts of the plant are highly nutritious and it serves as a substitute for soybean in the tropics because it can thrive in areas where the cultivation of soybean is difficult. In addition, its protein content is comparable to that of soybean.^19,20^ This crop is however faced with the problem of low yield. In recent times, the de novo transcriptome of winged bean was generated by Vatanparast et al.^21^ They identified Single Sequence Repeats (SSR) and Single Nucleotide Polymorphism (SNP) that can provide new resources for gene discovery and marker development. 18 SSR markers have also been validated for genetic diversity in winged bean by Wong et al.^22^ Further identification of trait-specific markers was studied by Chankaew et al.^23^ using linkage mapping. They discovered 31 QTLs controlling pod length, pod color, pod anthocyanin content, flower color, and seed color in winged bean. Despite the information obtained from these studies and several others, there is still a lack of trait-specific markers and improved varieties for yield.

### Zombi Pea (Vigna Vexillata (L.] A. Rich)

Zombi pea, an orphan legume is the least known of the domesticated Vigna spp. It is known to be of African origin and it exists in two forms (i.e. seeds and tubers (storage roots). Zombi pea is also grown in Southeast Asia, Australia, and India.^24,25^ It is believed that the tuber type originated in Asia, whereas the seed type originated in Africa.^25^ Compared to sweet potatoes and tapioca, the protein content of Zombi pea tubers was found to be up to eight times higher. The Vigna vexillata tuber is one of the domesticated Vigna species that has received the least attention in terms of genetic resource research, particularly in India. Despite their worth, collections from Africa have drawn greater interest than those from Southeast Asia, which includes India.^26^ The genetics of domestication traits in zombi pea were investigated using QTL analysis.^25,27^ Other QTLs detected in Zombi pea include those for salt tolerance,^28^ and bruchid resistance.^29^ More QTLs for abiotic and biotic stresses need to be identified to initiate marker-assisted selection for breeding programs.

### African Yam Bean (Sphenostylis Stenocarpa (Hochst. Ex A. Rich))

African yam bean (AYB) is a neglected legume that can also contribute toward alleviating food insecurity in sub-Saharan Africa.^30^ It is grown mainly for the nutritious seeds in western Africa while the tubers are utilized in eastern and central Africa. It is also a source of animal feed.^31^ AYB is consumed by some hypertensive, diabetic patients who have cardiovascular diseases in Nigeria.^32^ The low amount of sodium present in AYB seeds makes them suitable for consumption among hypertensive patients. Also, AYB is the right choice for diabetic patients because the rate of digestion is prolonged, avoiding the accumulation of sugar in the blood.^33^ The ability of African yam bean to be used during inter-cropping is of great benefit because it establishes nitrogen fixation in the soil.^34,35^ Reported that the lectins extracted from African yam bean are insecticidal. Recent findings revealed SNP alleles associated with some nutritional traits and seed size traits have been identified in AYB.^36,37^ Other studies recently carried out on AYB include (i) further studies to identify genetic diversity among some AYB accessions toward crop improvement;^36,38,39^ (ii) identification of diseases/infections such as bean common mosaic virus (BCMV) which limit the yield of AYB;^40^ (iii) mutation in AYB.^41–43^

Despite the amount of research that has been carried out on orphan crops, there is still a long way to go in developing better varieties of these crops without compromising their inherent ability to meet the standards of foods in the food system. Also, the conservation of orphan crop landraces is necessary for additional assessments, and modern breeding initiatives should be used to enhance promising landraces and their attributes.^38^ We have identified some research gaps (Table 1) for some orphan crops. If these gaps are addressed, then these crops can help in achieving food and nutritional security quickly.

**Table 1.** Some orphan crops, their potential trait for crop improvement, research gaps and genome sequence status.

Crop [Common name)	Scientific name	Potential	Genomic Markers	Genome Sequence Status	Chromosome Number	Genome Size	Breeding Effort	Available Genomic Tools	Availability Transformation system	Research Gap	References
Hyacinth bean	*Lablab purpureus* L.	Drought and salinity tolerance, nutritious, nutraceutical properties	EST-SSRs, RAPD, EST-SNP, InDel.	No	2 n = 2x = 24	367Mb	Improved photoperiod insensitive and development of determinate pureline varieties	Linkage map, QTL mapping	Nil	No superior cultivars and resistant varieties to pest and weeds	44,45
Zombi pea	*Vigna vexillata* [L.] A. Rich	Nutritious, biotic and abiotic stresses resistance	RAD-seq, SSR, SNP	No	2 n = 2x = 22	803Mb	Identification of candidate gene, VvTaXI for Bruchid resistance	QTL mapping	Nil	No improved zombi pea for abiotic stress resistance	24,46,47
African Yam Bean	[*Sphenostylis stenocarpa* [Hochst ex. A. Rich.] Harms]	Nutritious, versatile	AFLP, ISSR, SSR, SNP	77–81%	2 n = 2x = 22	> 649Mb	Identification of various agronomic traits	Association mapping	Nil	There is need to improve crop yield, shorten the cooking and maturity periods, and increase the crop’s resilience to pests and diseases.	48,39
Winged bean	*Psophocarpus tetragonolobus* (L.) DC.	Nutritious, versatile, adaptable to diverse soil types	AFLP, RAPD, SSR, SNP,	98.9%	2 n = 2x = 18	1.22Gb	Identification of quantitative trait loci [QTLs) for traits such as pod and seed characteristics	Cloning, transcriptomics, Genetic linkage maps	Nil	Little or no information on how to improve the long maturation period, indeterminate growth habit, low seed yield, and the need for a stake to support the vigorously growing vines	19,49
Bambara groundnut	*Vigna subterranea* [L.] Verdc	Nutritious, climate-resilient, versatile, adaptable to diverse environment	ISSR, SSR, SNP	No	2 n 2x = 22	550Mb	Identification of QTL for some agronomic traits	Whole Genome Sequencing, Transcriptome sequencing	Nil	No improved line for yield and adaptation to moisture stress	44,50,51
Finger millet	*Eleusine coracana*	Resilience to adverse climatic conditions, high nutritional content, versatile	EST-SSR, ISSR, SSR, SNP	100%	2 n = 4x = 36	1.45Gb	Development of lines with improved agronomic traits	Linkage mapping, QTL mapping, Whole Genome sequencing, Reference genome, Transcriptomic, Proteomics, RNA-Seq	Agrobacterium-mediated transformation and particle bombardment [biolistics]	Identification of novel genes for abiotic stress tolerance and nutritional traits	52,53
Chickpea	*Cicer arietinum*	Nutritious, adaptability to diverse climates	AFLP, ISSR, InDel, ISM, ILP, SSR, SNP	73.8%	2 n = 2x = 16	738Mb	Identified loci associated with yield, drought tolerance, and resistance to diseases	Linkage mapping, QTL mapping, GWAS, Whole Genome sequencing, Reference genome, Proteomics, RNA-Seq, DGE, CRISPR/Cas9	Agrobacterium-mediated transformation and particle bombardment [biolistics]	Selection of accessions that can be used as parental material in breeding programs. ^Lack of drought-tolerant cultivars.^	54,55
Foxtail millet	*Setaria italica*	High nutritional content, environmental resilience, and potential health benefits	KASP, AFLP, RAPD, InDel, SSR, SNP	100%	2 n = 2x = 18	490Mb	Identification of genetic loci associated with drought resistance, plant height, flowering time, grain yield, and nutritional quality	Linkage mapping, QTL mapping, GWAS, Whole genome Sequencing, Comparative Genomics, CRISPR Cas9, DGE, TILLING, MAS, GS	Agrobacterium-mediated transformation and particle bombardment [biolistics]	Lack of high yielding varieties	56,57
Grass pea	*Lathyrus sativus*	Ability to thrive in adverse conditions, high nutritional value	AFLP, RAPD, EST-SSR, KASP, SSR, SNP	100%	2 n = 2x = 14	6.3Gbp	^Identification of^ stress-responsive genes	Draft Genome Assemblies, Transcriptome sequencing [RNA-Seq], Comparative Genomics, Gene Knockout, TILLING	^Nil^	Lack of genetic improvement through GWAS and MAS	58,59
Horsegram	*Macrotyloma uniforum*	Nutritional and therapeutic properties, resilience to harsh environmental conditions	RAPD, ISSR, SSR, SNP, ILP, COS	83.53%	2 n = 2x = 20 or 22	400Mb	Identification of genes involved in metabolic process, Identification of candidate gene involved in environmental adaptation	Linkage mapping, QTL mapping, Whole Genome Sequence, Transcriptome sequencing, Comparative Genomics, GWAS, DEG	^Nil^	Candidate genes underlying functional traits has not been identified	60,61
Pigeon pea	*Cajanus cajan*	Nutritious, versatile, nutraceutical properties, adaptability to marginal environments, drought resistance	SSAP, REMAP, SCAO, SSR, SNP	100%	2 n 2x = 22	833Mb	Identification of regions associated with Fusarium wilt resistance, sterility mosaic disease resistance, drought tolerance, and pod yield	Whole Genome sequence, GWAS, MAS, Transcriptomics, Comparative Genomics, CRISPR	Agrobacterium-mediated transformation	Lack of breeding for high yielding pure line and hybrid	62,63
Moth bean	*Vigna aconitifolia*	Nutritious, adaptability to harsh climates, nutraceutical properties	AFLP, RAPD, ISSR, SSR, SNP	50%	2 n 2x = 22	409Mb	Identification of stress-responsive genes	Linkage mapping, Proteomics, Comparative Genomics,	Nil	Lack of reference genome, little integration of modern genomics tools, Lack of common features of nutritional, anti-nutritional compounds, processing characteristics, and health benefits	64,65
Black gram	*Vigna mungo*	High protein content, adaptability to various cropping systems	AFLP, RAPD, ISSR , SSR, SNP	79.2%	2 n = 2x = 22	574Mb	Identification of SNPs associated with seed size, flowering time, and biotic stress resistance	Draft Genome Assembly, Linkage mapping, GWAS, QTL mapping, DEGs	Agrobacterium-mediated transformation	Incomplete reference genome, lack of high yielding and MYMV resistant varieties	66,67
Adzuki bean	*Vigna angularis*	Nutritious, functional food and medicinal applications	AFLP, ISSR, EST-SSR, SSR, SNP	97.8%	2 n = 2x = 22	542Mb	Identification of SNPs associated with seed size, flowering time, and disease resistance	Draft Genome Assembly, Linkage mapping, GWAS, QTL mapping, DEGs, Comparative Genomics, MAS	Agrobacterium-mediated transformation	Little knowledge on utilization. Little understanding of the anti-obesity mechanism and the specific bioactive components involved has not been identified.	68–70
Mung bean	*Vigna radiata*	Nutritious, nutraceutical properties, drought tolerance, disease resistance, climate-resilient	AFLP, RAPD, ISSR, SSR, SNP	96.9%	2 n = 2x = 22	543Mb	Identification of SNPs associated with seed size, flowering time, and disease resistance	Draft Genome Assembly, Linkage mapping, GWAS, QTL mapping, DEGs, Comparative Genomics, MAS	Agrobacterium-mediated transformation	Little knowledge on the unraveling of the main functional components relevant to health benefits	71,72
Fonio Millet	*Digitaria exilis*	Fast maturing, climate-resilient, nutritious, nutraceutical properties, highly adaptable to poor soils and drought conditions	AFLP, RAPD, SSR, SNP	99%	2 n = 4x = 36	761Mb	Identification of drought resistance traits	GWAS, DEGs, Comparative Genomics	Nil	Improvement and domestication is lacking	73

## Research strategies for improvement

There are ongoing efforts to see orphan crops together with major crops in the global food basket. However, there is a need for more strategies that can speed up the improvement of orphan crops to remove the inherent natural barrier that causes them to stay orphan not just from conventional plant breeders alone, but also from biochemists, plant geneticists, botanists, and traditional farmers.^74^ The use of high-throughput phenotyping such as Ground Based Imaging, Unmanned Aerial Vehicle (UAV) imaging, and satellite imaging has shown promises in increasing the speed of genetic gain by the precise measurements of desired traits among thousands of field-grown plants within a short period of time.^75^ The development of core collections is another strategy for accelerating improvement in orphan crops as it serves the objective of supplying data required to enhance the utilization of genetic resources in any crop improvement program.^76^ It eliminates the time it takes breeders to estimate genetic variability, evaluate germplasm, and select parents for crop improvement, and also the development of a core collection allows for target allele mining.^77^ Exploring the genomics of these orphan crops via conventional breeding, Genome-Wide Association Studies (GWAS), modern biotechnology, and computational technology will also hasten orphan crop improvement for wide acceptability and utilization.^74^

Several newly developed technologies and breeding strategies that enable the swift identification and selection of genes underlying desirable traits thereby shortening the time for selection can be applied to fast-track the improvement of orphan crops.^78^ Modern technologies in genomics and gene editing like the Clustered Regularly Interspaced Short Palindromic Repeats (CRISPR/Cas system), Transcription Activator-Like Effector Nucleases. (TALENs) and Zinc-Finger Nucleases (ZFNs) can be used to enhance the productivity of orphan crops. However, these techniques rely on the sequenced information of the crop. Sequencing the genomes of different orphan crops with tools such as Genotype by Sequencing (GBS) and Single Nucleotide Polymorphism (SNP) will provide the information needed to use these modern technologies to hasten crop improvement. Genes identified from sequencing can then be designed for enhancement using these modern technologies.^79^ Domestication of orphan crops is another method for hastening their improvement. This can lead to the faster improvement and utilization of the crops. Overall, the use of all of these methods in a well-structured breeding program for orphan crops can contribute to their rapid improvement.

## High-throughput phenotyping

Over the years a variety of high-throughput phenomics methods have been used for phenotyping trait-specific characters including growth, phenology, physiology, disease incidence, insect damage, and drought tolerance in major crops, this has accelerated the period between breeding cycles in these crops.^80^ This can also be replicated in orphan crops to provide the same advantage ^81^ since it is evident that orphan crops will need more efficient phenotyping to dissect important traits they possess. Not only does high-throughput phenotyping hasten the breeding cycle it also enables the fast and accurate measurements of many important traits in model and non-model plant species. For example, the use of high-throughput phenotyping of field-grown cassava in the ongoing ‘cassava source-sink project’ will hopefully overcome some of the limitations of single-trait engineering which will lead to the rapid enhancement of cassava as an orphan crop.^82^ In addition, images of more than one thousand sorghum (*Sorghum bicolor*) panicles from 272 genotypes comprising a subset of the Sorghum Association Panel were used to create a phenotyping pipeline that extracted unidimensional and multidimensional features from the images. This high-throughput phenotyping tool can be used to collect phenotypic data from large orphan cereal populations, causing a transformation in their genetic improvement.^83^ More so Großkinsky et al.,^84^ in combining multi-omics and advanced phenotyping discussed how these techniques can facilitate the generation of climate-smart *Camelina sativa* (camelina, gold-of-pleasure, false flax) – an orphan oilseed crop.

Zhang et al.^85^ demonstrated that under active field conditions images extracted from multispectral cameras to estimate canopy area, percentage of canopy area, and vegetation indices and images from a thermal camera used to estimate mean canopy temperature can be used for monitoring Ascochyta blight disease severity in chickpea. They concluded that real-time information about traits acquired from high-throughput phenotyping techniques can assist efficient, accurate, and timely decision-making for crop improvement. In addition, a high-throughput phenotyping protocol to screen lentil accessions for salt tolerance was developed by Dissanayake et al.^86^ This protocol can be used to screen a large number of lentil accessions in field conditions for salt tolerance. The use of spectroscopic technique FT-MIR coupled with attenuated total internal reflectance sampling interface to develop multivariate models for total protein concentration in chickpea (*Cicer arietinum* L.], dry pea (*Pisum sativum* L.), and lentil [*Lens culinaris* Medik) was demonstrated by Madurapperumage et al.^87^ They found out that FT-MIR spectroscopy can quantify protein traits quickly and easily in orphan pulse crops. Since the use of techniques such as Marker-Assisted Selection and genome-wide association mapping depends on accurate phenotypic data, the use of high-throughput phenotypic techniques on orphan crops can lead to the accurate detection of markers underlying phenotypic traits.

## Marker assisted breeding

This method of breeding is extensively utilized in staple crops and has begun to be employed in some of the orphan crops including barley,^88^ cassava,^89^ pearl millets,^90^ peanuts,^91^ and some perennial plants.^92^ The development of molecular markers was once a time-consuming and labor-intensive process. However, the advent of next-generation sequencing (NGS) has enabled the development of large-scale molecular markers, including microsatellite or simple sequence repeat (SSR), insertion-deletions (InDels), and single nucleotide polymorphisms (SNP), this has also made the turnaround time for crop improvement faster.^92^ The identification of molecular markers underpinning phenotypic traits through studies such as GWAS, and bi-parental QTL explains the genetic basis of such traits making the manipulation of the trait faster and easier thereby enabling the development of new varieties via marker-assisted breeding. ^93^

## Whole genome sequencing

Next-generation sequencing (NGS), which allows for the sequencing of the whole genome of many crops and their wild relatives, is an efficient way to facilitate the domestication of desired genes in orphan crops, thereby hastening their improvement.^78^ Over the years, as scientific research has shifted focus to orphan crops, whole genome sequences of some orphan crops have been carried out. Genome sequencing has enabled the identification of upstream regulatory elements of genes and the fine-tuning of the expression of target genes, and all these are possible due to the advent of NGS approaches. These approaches have made it possible to quickly identify target genes and manipulate such genes to bring about new improved crop varieties in a shorter period. A number of orphan crops’ whole genome has been sequenced (Table 1). Zhang et al.^94^ sequenced the genome of foxtail millet and reported that around 1500 genes are unique to this crop, where about 580 genes were marked as ‘response to water.’ Reannotation of these genes provided insights into their classification, wherein a few stress-responsive genes were characterized at a genome-wide level.,^65^ developed a de novo genome assembly of *Vigna aconitifolia* using PacBio High-Fidelity reads and Hi-C sequencing data, with a total size of 409 Mb and contig N50 of more than 30Mb. The genome was also annotated for repeat sequences and they found out that the moth bean genome comprises about 54% of repetitive sequences, and predicted 36950 protein-coding genes. The identification of these genes can lead to the rapid genetic improvement of this crop.

## TILLING (Targeting induced local lesions in genomes) and Eco-TILLING

These are effective methods for hastening orphan crop improvement since the techniques do not require biosafety regulations as they are free of transgenes. TILLING is a reverse genetic technique that uses traditional mutagenesis followed by high-throughput mutation detection. It is applied to the induced mutagenized population while Eco-TILLING is applied to the natural population.^95^ Eco-TILLING was used to identify single nucleotide polymorphisms (SNPs) and small insertions/deletions (INDELS) in a collection of mung bean accessions. A total of 157 DNA polymorphisms in the collection were discovered in the collection of limited diversity.^96^ Desi and Kabuli chickpea accessions are known to have low genetic polymorphism. However, employing eco-TILLING found approximately 31% phenotypic variation for 100 seed weights.^97^ TILLING and Eco-TILLING have been applied to very few orphan crops to access their diversity but are yet to be used on several orphan crops in inducing mutagenized populations for their improvement.

## Genome-wide association studies (GWAS)

Genome-wide association studies (GWAS) are a crucial marker-trait association (MTA) study for accelerating crop improvement.^98^ This method identifies Quantitative Trait Loci (QTLs) from high-density SNP maps that are linked to phenotypic traits in crops. These SNPs can be used to identify the genetic basis of adaptive traits in orphan crops and can be designed and used for Marker Assisted Breeding. Candidate genes of desired traits obtained from orphan crops using GWAS can be used to improve the crop using a genetic modification-type approach.^2^ While thousands of these methods have been reported for staple crops, GWAS of orphan crops is still in its early stages.^2^ For instance, there is little or no record of Genome-Wide Association studies for winged bean. However, it has been carried out on some orphan crops for some traits. By employing DArTseq SNP for a Genome-Wide Association Study, Quantitative Trait Loci (QTL) that could be useful for the improvement of the protein, oil, and starch contents of African Yam Bean were identified by Oluwole et al. and Olomitutu et al.^36,37^

In addition, in mung bean, Genotype-by-Sequencing GWAS identified 116 SNPs in 61 protein-coding genes. Sixteen of these protein-coding genes have been found to enhance phosphorous uptake and utilization efficiency. Six of these genes showed high expression in the root, shoot apical meristem and leaf. The SNPs in three of these genes have been validated using a Sanger sequencing approach.^99^ Also, on mungbean, Xu et al.^100^ identified 19 QTLs containing 32 SNPs that were significantly associated with alkali tolerance on nine chromosomes at the germination stage. Using GWAS, Uba et al.^98^ identified a total of 27 significant marker-trait associations (MTAs) for seed color, days to flowering, days to maturity, terminal leaf length, terminal leaf width, number of seed per pod, pod width in Bambara groundnut. A total of 17 candidate genes were identified, varying in number for different traits for the above-mentioned traits. Marker-trait association analysis in moth bean revealed 29 potential genomic regions for the trait days to 50% flowering.^101^

## Speed breeding

The timing of the release of genotypes to farmers has a considerable impact on breeding. In conventional breeding, the breeding cycle takes roughly three to seven years, while the yield, disease, and quality tests take about four years, and the variety release takes an additional one to three years. Long-term improvements are made to a single gene or variety. As a result, a technique known as “speed breeding” has been devised, which involves altering day-light and duration in order to abbreviate the life cycle.^102^ It also enables rapid generation advancement by growing plant populations under controlled photoperiod and temperature regimes to hasten their growth and development. It can play a role in the rapid development of improved crop varieties supporting the cultivation, production, and utilization of orphan crops at a large scale. Using this technology, there was a significant reduction in the time taken for anthesis to occur in spring wheat (*Triticum aestivum*), durum wheat (*Triticum durum*), barley (*Hordeum vulgare*), Chickpea (*Cicer arietinum*), and canola (*Brassica napus*) planted in a temperature-controlled glasshouse fitted with high-pressure sodium lamps. This significantly increased the number of generations grown per year of these crops.^103^

Samineni et al.^104^ developed a protocol for increasing the number of generation cycles per year in chickpeas using speed breeding. Their results showed encouraging implications for breeding programs as they were able to produce 7, 6.2, and 6 generations per year in early-, medium-, and late-maturing accessions. This makes for rapid progression toward homozygosity, development of mapping populations, and reduction in time, space, and resources in cultivar development. This technology is important for methods like genomic selection as it reduces the time it takes to generate one breeding cycle.^78^ Plant breeders and plant molecular geneticists can use the speed breeding technique to quicken the genetic variety present in wild relatives of these orphan legumes, introducing top variants that will be widely accepted and cultivated by farmers.

## CRISPR/Cas

Clustered regularly interspaced short palindromic repeats (CRISPR)/CRISPR-associated proteins (Cas) is the most straightforward, adaptable, and precise strategy for genetic manipulation in plants. Genetic manipulation such as gene knockout, promoter editing, gene knock-in, base editing, and prime editing using the CRISPR/Cas9-assisted gene editing technology has been applied to major crops with success rates some of which include *Medicago truncatula* and soybean,^105^ cowpea,^106^ rice,^107^ cassava,^108^ wheat,^109^ tomatoes,^110^ and alfalfa.^111^ CRISPR/Cas9-assisted gene editing technology has also been applied to some orphan crops with a success rate. Phytoene desaturase (PDS) knockout has been achieved in the first application of CRISPR in foxtail millet (*Setaria italica*) protoplasts. This indicates that the technology can be used to improve millet.^112^

The CRISPR/Cas9 technology is also the only Site-Specific Nuclease (SSN) to allow efficient multiplexing – a method for simultaneously targeting multiple genes/alleles by multiple using gRNAs. Multiplexing with CRISPR/Cas has been demonstrated in major crops such as soybean ^113^ and maize ^114^ as well as in some orphan crops such as Eggplant,^115^ foxtail millet ^116^ and Chickpea.^117^ In addition, by utilizing CRISPR/Cas9 single- and multi-gene knockout systems to target SiALS and SiACC genes in foxtail millet protoplasts,^116^ developed a homozygous herbicide-tolerant mutant plant using the Cytosine Based Editor to target SiALS gene. Furthermore, Badhan et al.^117^ demonstrated the knockout of 4-coumarate ligase (4CL) and Reveille 7 (RVE7) genes in the chickpea protoplast using DNA-free CRISPR/Cas9 editing. High-efficiency editing was achieved for the RVE7 gene in vivo compared to the 4CL gene. This reveals that CRISPR/Cas9 can be used to target agronomically important traits in chickpea.

The success of this technology in these crops shows promise for its application in other orphan crops to target traits beneficial to farmers and consumers. However, in-depth bioinformatic, genomic, and transcriptomic information, and optimization methods for the transformation and regeneration of orphan crops are important for their improvement as the technology relies on characterized functional genes and efficient transformation and regeneration protocols.^118^ Therefore, information from studies such as whole genome sequencing, QTL, GWAS, marker-assisted selection, genetic transformation, and regeneration in orphan crops is crucial.^74^ Advantageously, the genome of some orphan crops such as African yam bean and Adzuki bean is being sequenced, and that finger millet assembled by the AOCCC^119^ and the genome of pigeon pea has been sequenced by Varshney et al.^62^ and white fonio millet by Wang et al.^73^ Information obtained from these sequences can be used to acquire genomic targets for designing gRNAs for CRISPR/Cas gene editing in these crops. As demonstrated in major crops and some orphan crops, improvement of orphan crops using the CRISPR/Cas technology is achievable.^118^

## Developmental strategies for improvement

### Market development

There is growing global interest in new foods and products that can enhance health and nutrition. This trend presents an opportunity to develop markets for non-staple crops, potentially benefiting impoverished communities.^120^ Market, commercialization, and demand limitations are key aspects in promoting any orphan crop. These limitations are often due to the stigma of food of the poor that often accompanies these traditional crops in any country. Promoting the nutritional benefits of orphan crops through consumer education, organizing cooking demonstrations and workshops to showcase their versatility, and developing clear labeling and certification standards can build consumer trust and increase preference for these crops, thereby boosting their production.^121^ Popularizing consumption and enhancing market image through partnerships with food companies can address these challenges, as demonstrated by Andean grains. For example, a partnership with the Bolivian coffee shop chain “Alexander Coffee” promoted these grains through marketing campaigns and innovative food recipes, directly linking farmers to the chain for raw material supply.^122^ New food products incorporating Andean grains were developed, and their nutritional benefits were promoted to increase consumption and inclusion in government programs.^123^

Strengthening all stages of the value chain from production to consumption and ensuring efficient processing, marketing, and distribution are important to place orphan crop products on supermarket shelves. Value addition is another strategy that can be employed to enhance the acceptability of orphan crops. This process involves economically enhancing the value of a product by altering its characteristics to align with marketplace preferences.^122^ In India, adding value to millet nearly tripled farmer incomes and generated new employment opportunities, particularly for women.^120^ Establishing robust market linkages between producers and various markets, including local, regional, and international ones, is crucial for commercializing orphan crops. This involves conducting market studies to identify potential markets and consumer preferences for orphan crop products and establishing efficient supply chains to connect farmers with these markets and ensure timely product delivery.^7^

### Policy and institutional support

Agricultural policies and institutions play a crucial role in crop development by providing support, guidance, and resources to farmers and agribusinesses. These policies and institutions help promote sustainable agricultural practices, enhancing research and development, and ensuring the availability of necessary resources for crop production.^124^ For instance, national agricultural research systems in the developing world have been instrumental in the development and diffusion of modern crop varieties, contributing significantly to the Green Revolutions observed across various food crops such as wheat, rice, maize, and others.^125^ The United Nations General Assembly (UNGA) conventions have identified several developmental strategies to transform production systems, including: increasing research investments to explore the climate resilience and nutritional characteristics of underused and orphan crop species; providing greater support for their inclusion in food security policies and programs; encouraging their use to diversify farming systems and create more biodiverse landscapes and healthier ecosystems; and upgrading their value chains and markets to ensure their sustainable use.^8,126^

Various national and international research, funding agencies, and policy institutions, such as Consultative Group for International Agricultural Research (CGIAR), which comprises of institutes like the International Crops Research Institute for the Semi-Arid Tropics (ICRISAT), International Center for Tropical Agriculture (CIAT), International Institute of Tropical Agriculture (IITA), International Maize and Wheat Improvement Center (CIMMYT), International Food Policy Research Institute (IFPRI) and International Potatoes Center (CIP), Biodiversity International, African Orphan Crops Consortium (AOCC), Global Crop Diversity Trust, Svalbard Global Seed Vault, Crops for the Future (CFF), Food and Agriculture Organization (FAO), and the National Agricultural Research Systems (NARS) such as the Indian Council of Agricultural Research (ICAR), Kenya Agricultural and Livestock Research Organization (KALRO), Ethiopian Institute of Agricultural Research (EIAR) and National Root Crops Research Institute (NRCRI), Bill & Melinda Gates Foundation, McKnight Foundation, and Biotechnology and Biological Sciences Research and International Fund for Agricultural Development (IFAD) are actively working to identify best management practices and provide financial support for orphan crops.^5^

Despite these players, orphan crops still lack sufficient policy support, breeding programs, and funding. Crops like millet, sorghum, and teff often lack national policies promoting their cultivation and consumption despite their nutritional benefits and resilience to climate change.^127^ In Africa, funding and research focus heavily on maize and rice, while indigenous crops like fonio and bambara groundnut receive minimal attention.^127^ In countries like Nigeria, while cassava has received significant attention, other nutrient-rich orphan crops like African yam bean and moringa are often left out of major agricultural development programs. The implementation of policies, funding, and support that incentivize orphan crop production and processing could hasten their commercialization.

**Figure 1.**
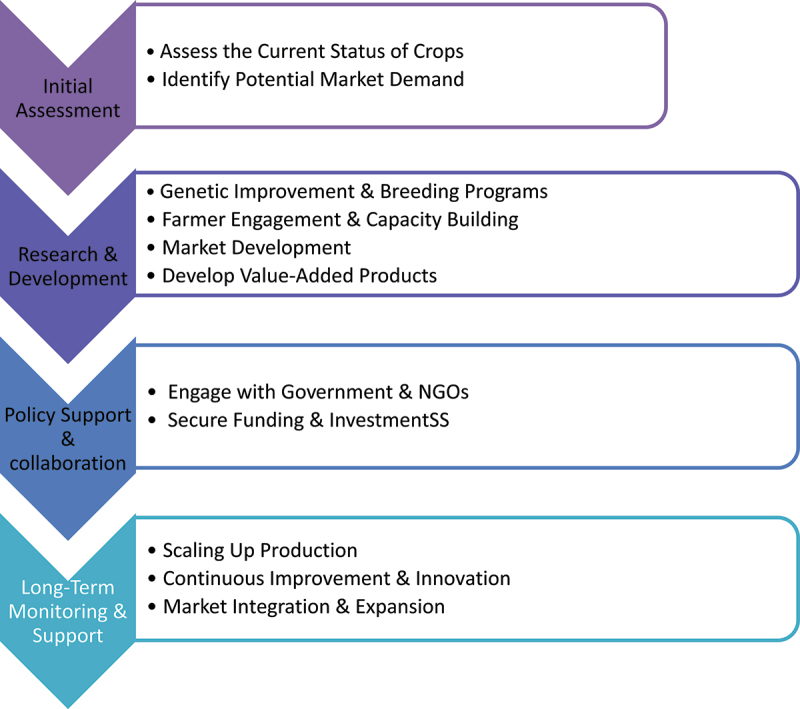
Transforming Orphan crops to a major crop.

### Capacity building

Intensive capacity-building efforts should be implemented to train farmers on the nutritional benefits, best cultivation practices, and marketing of orphan crops. This includes producing quality seeds, managing pests and diseases, and improving harvest and post-harvest methods. Additionally, these efforts should enhance farmers’ skills in novel food preparations, transformation processes, nutrition, food safety, and marketing.^122^ Training farmers in storage technologies to minimize post-harvest loss, as well as in processing technologies to convert orphan crops into value-added products, can enhance their marketability and shelf life. This, in turn, can accelerate the integration of orphan crops into the food system.^123^ To support orphan crop development, it is essential to enhance the capacity of local research institutions through training, funding, and partnerships with international research organizations. Facilitating knowledge exchange among stakeholders through conferences, workshops, and publications, along with investing in research facilities, laboratories, and processing plants, will further strengthen infrastructure and support the overall development of orphan crops.^8^

### Collaborative efforts

This involves the sharing of knowledge, resources, and technologies between countries, international organizations, governments, research institutions, and the private sector to tackle common challenges in crop improvement. Models such as Participatory Plant Breeding (PPB) could be utilized to facilitate the smooth collaboration between stakeholders. This breeding model involves farmers, government, private companies, research institutes, consumers, and other stakeholders in breeding improved crop varieties and ensuring that new varieties meet the needs and preferences of all those involved.^128^ This comprises sharing knowledge, making decisions, and pooling resources throughout the entire process of breeding crops, from initial planning to product launch. By understanding the needs and preferences of these groups, developers can create crop varieties that meet market demands with high adoption rates, improve farmers’ livelihoods, and contribute to food security.^129^ The PPB approach integrates biological, psychological, and social dimensions, proving effective in promoting food sovereignty.^130^

In the late 1990s, the International Center for Agricultural Research in the Dry Areas (ICARDA) developed the first participatory action research projects for plant breeding in Morocco, Syria, and Tunisia. These projects were initiated due to the low yield of staple crops, high malnutrition, and famine risk in these regions, where conventional breeding methods failed to address farmers’ needs. Decentralized and participatory plant breeding (PPB) proved effective by involving farmers from the beginning, ensuring crops suited to local contexts and farmers’ knowledge.^128^ Farmers’ involvement in selection hastened the adoption of new varieties.^131^

PPB method has been especially useful for maize growers in the USA and Portugal and farmers’ input was central to the success of the program. The US project was an equal partnership between farmers and researchers from the start, while the Portuguese project began with researchers who later sought farmer collaboration. In the US, the ‘Who gets kissed?’ project was initiated by an organic vegetable grower and a scientist from a nonprofit research institute, later joined by a public sector university plant breeder. The project aimed to develop an open-pollinated sweet maize variety under organic systems. Farmers and researchers collaboratively evaluated quality, making the breeding process more social than traditional methods. They completed five cycles of selection and, in 2014, released the variety ‘Who Gets Kissed?’ allowing regional breeding projects to adapt it to local conditions.^132^

Bread wheat (*Triticum aestivum*) has numerous participatory breeding (PPB) projects, often stemming from farmers’ needs for organic and artisanal bread-making varieties. Key motivations include unmet farmer needs, genotype-by-environment interactions, and logistics. PPB projects involve decentralized selection and addressing issues like Fusarium and pre-harvest sprouting, with farmers and researchers collaborating. Successful projects show that farmer-developed varieties can yield equivalent to modern varieties with added desirable traits and more excellent stability. Market acceptance is crucial for farmers, who often focus on value-added production through local mills and bakeries.^132^

In the Netherlands, farmer-breeders worked closely with commercial breeding companies for collaborative potato breeding. The model was successful and contributed significantly to developing a diverse portfolio of potato varieties for the national and international markets.^133^

PPB has also been implemented in developing countries where farmers with limited resources grow their crops in marginal lands of remote regions. It is also implemented in areas where the technology transfer or adoption of modern cultivars is low (as farmers are not comfortable with taking the risk of replacing their well-known and reliable traditional varieties with new varieties) or where modern cultivars are not available, this is the case for *orphan crops*.^134^

Participatory Plant Breeding (PPB) was implemented for Pearl millet (*Pennisetum glaucum*) and sorghum (*Sorghum bicolor*), both considered orphan crops, by ICRISAT in Mali, Niger, and Burkina Faso to develop new varieties through farmer participation. Participatory variety evaluation trials allowed farmers to test and select promising varieties, leading to better yield performance and early maturity. Furthermore, seed production and commercialization were facilitated through collaboration with farmer organizations, seed fairs, and emerging private seed enterprises, ensuring the availability of improved crop varieties in the market.^123^

In Peru and Bolivia, IFAD funded a project to enhance the use of orphan crops, focusing on the case of Andean grains (quinoa, cañahua, and amaranth). Improved varieties of quinoa, cañahua, and amaranth were developed and disseminated using methods, including the PPB method. The project had a positive impact on the production, conservation, and income generation of Andean grains.^123^

Linking orphan crop breeding to market and value chain development offers considerable potential for their development, and PPB programs could prove useful for hastening the improvement and commercialization of orphan crops.^134^ A flow chart summarizing research and developmental strategies for hastening orphan crop improvement is presented in Fig. 1.

### Role of the genebanks

Approximately 1% of accessions conserved in different global germplasm repositories have been exploited for crop improvements. The use of plant genetic resources for developing new varieties has not changed much since 2006, according to reports from the FAO.^135^ Few success stories have been reported regarding the use of alleles from traditional landraces in elite breeding programs,^136^ likely due to the lack of knowledge regarding the potential usefulness of conserved germplasm and the capacity to identify and transfer useful alleles to improved varieties.^137^

The cultivation and conservation of orphan crops contribute significantly to biodiversity maintenance, food and nutritional security, and the livelihoods of small-scale farmers by offering diverse income opportunities.^79^ Genebanks conserve plant genetic resources, including wild relatives or landraces of orphan crop species that possess wide genetic diversity and are reservoirs of useful genes.^81^ These genes have occasionally been instrumental in improving elite species. For instance, dwarfing genes from East Asian rice and wheat landraces were successfully introgressed into elite rice and wheat during the Green Revolution. Additionally, Ethiopian landrace barley contains mlo alleles, conferring broad-spectrum resistance to powdery mildew, which have been introgressed into elite barley.^136^

Most genebanks conserve a large proportion of minor, neglected, and orphan crops that lack sufficient genomic resources but are repositories of valuable traits for breeders.^137,138^ The initial step for genebanks to hasten orphan crop improvement involves comprehensive and precise genotype-to-phenotype mapping for each accession.^136,139^ This genetic characterization provides insights into traits such as tolerance to biotic and abiotic stresses, disease resistance, yield potential, improved nutritional profiles, and unique phytochemical compounds, enabling informed decisions in breeding programs (FAO, 2015). Multi-location trials are essential to assess accession performance across diverse environmental conditions. This enables genebanks to identify superior genotypes with desirable agronomic and quality traits, informing the selection of parental breeding lines and the development of pre-breeding populations.^77^

Genebanks should aim to provide comprehensive knowledge of germplasm accessions, their relationships, descriptive characters, and optimal evaluation methods. They should adopt genotypic-based plant identification to establish well-resolved phylogenetic relationships between cultivated species and their Crop Wild Relatives, aiding gene discovery and defining strategies for crop improvement.^137^ Additionally, core collections based on genetic variation and specific traits should be developed for orphan crops to enhance their use in improvement programs. High-throughput phenotyping combined with genotyping should be employed to study functional genetic variation and discover markers for economically important traits efficiently.^138^ Allele mining, including methods like eGWAS, should be applied to all orphan crops to uncover alleles for climate resilience, prioritizing their conservation and use in breeding resilient crops. This information from genebanks serves as a foundation for breeders to expedite orphan crop improvement efforts.^137^

## Case studies and success stories of orphan crop improvement

### Finger Millet (Eleusine Coracana)

Finger millet, a staple food in East Africa and South Asia, exemplifies successful efforts in improving and commercializing orphan crops.^120^ It is known for its climate resilience against drought and is valued for its nutritional properties and health benefits. Despite these advantages, the cultivation area of minor millets like finger millet declined globally, with an annual production hovering around 3 million tonnes due to challenges such as pests, diseases, striga weed, poor soil quality, suboptimal agronomic practices, and inadequate product development strategies.^120,140^

Breeding programs of finger millet prioritized enhancing yield potential, disease resistance, and market appeal ^141^ and through targeted breeding and genomic tools integration, improved finger millet varieties have been developed to meet yield and dietary requirements, resist diseases, and attract commercial interest in gluten-free and functional food markets.^142^ Interspecific hybridization involving Indian and African germplasm has further enhanced yield and disease resistance while transgenic varieties have been developed for resistance against leaf blast disease, drought, and salinity stress.^143^

Recent collaborative efforts among international and national research institutions in India, several African countries, Nepal, and Sri Lanka – such as ICAR, KALRO, NaSARRI, EIAR, ICRISAT, and ICARDA – have focused on developing new and improved finger millet cultivars. This global collaboration has resulted in the registration of 143 advanced cultivars by ICRISAT, which now benefits farmers in Kenya and India, with reported yields of up to 10 tons per hectare. These advancements are not just improving farmer incomes and health but also contribute to the shared mission of sustainable agriculture.^144^ The recent completion of finger millet genome sequencing is a significant milestone in enhancing future breeding and improvement efforts.^145^

### Quinoa (Chenopodium Quinoa)

Once regarded as a traditional ‘peasant food,’ quinoa has remarkably evolved into a globally traded commodity. This transformation is mainly due to its status as a ‘superfood,’ celebrated for its exceptional nutritional content and adaptability to diverse growing conditions. Its superior nutritional qualities and the growing demand for healthy, gluten-free foods have been key drivers of its worldwide popularity. However, local quinoa varieties typically have low yields and are prone to lodging, shattering, and pest infestations.^146^ In response to the high demand for quinoa, research initiatives have aimed at enhancing yield, disease resistance, and processing characteristics, leading to the release of improved varieties.^147^ Furthermore, the commercialization of quinoa has been greatly influenced by genetic advancements, market development strategies, and the establishment of international supply chains. For example, the United Nations’ declaration of 2013 as the “International Year of Quinoa” significantly raised global awareness about the crop’s nutritional and cultural importance. This initiative, along with marketing campaigns, improved agronomic practices, and international trade agreements, facilitated quinoa’s entry into global markets.^148^ Moreover, the commercialization of quinoa has had notable socioeconomic impacts both in the Andean region and globally. For smallholder farmers in the Andes, the increased demand for quinoa has resulted in higher incomes and improved livelihoods.^149^

### Chickpea (Cicer Arietinum L.)

Before its genetic and genomic resources were available in 2005, chickpea – currently ranked as the second most significant legume in the world – was considered an orphan crop.^150,151^ Chickpeas are generally grown by rural farmers in dry and semi-arid regions. However, due to their nutritional benefits, socioeconomic importance, and ability to adapt to a variety of climatic conditions, chickpea popularity has increased.^152^ Furthermore, through scientific research, production techniques, marketing tactics, value addition, and international collaboration, this native crop has developed into a global crop.^153^ In recent times, scientific research has made it possible for chickpeas to undergo substantial genetic advancements, which have led to improvements in their nutritional quality, disease resistance, and productivity.^154^ Organizations such as the ICRISAT, ICARDA, and National Agricultural Research Centers, with funding from organizations such as the FAO, IFAD, and Bill and Melinda Gates Foundation, managed breeding programs that led to the development of improved varieties, which aided its commercialization.^150^

Presently, there are over 200 improved varieties of chickpea^151^ and the adoption of these improved varieties has significantly increased the income of chickpea farmers thereby reducing household poverty.^155^ The availability of draft genome assemblies, comprehensive genetic and physical maps, a vast number of SSR and SNP markers, millions of SNPs, and various high-throughput and low-cost marker genotyping platforms of chickpea has led to the development of superior lines. These lines exhibit enhanced drought tolerance, and resistance to Fusarium wilt and Ascochyta blight, making their way to both farmers’ fields and markets.^151^ Chickpea is now being cultivated in over 57 countries with India, Australia, Canada, Turkey, and Mexico being the top producers and exporters.^156^ Consumers increasing demand for plant-based proteins, awareness of the nutritional benefits of chickpea and the utilization of chickpea protein to produce several chickpea-based products such as hummus, chickpea flour, milk, butter, and snacks have also significantly influenced the extent of its market reach.^157^ The advancement of chickpeas from local staple food to a widely cultivated crop through breeding initiatives, product diversification, and consumer awareness offers vital insights for improving and commercialization of other orphan crops.^158^

### Bambara Groundnut (Vigna Subterranea)

Bambara groundnut, native to sub-Saharan Africa, has gained recognition for its nutritional value, drought tolerance, ability to improve soil fertility through nitrogen fixation, and potential for sustainable agriculture.^159,160^ Though an orphan crop, through collaborative efforts, bambara groundnut has experienced a remarkable transformation over the years.^159^ The success of Bambara groundnut improvement can be attributed to the establishment of the International Bambara Groundnut Network (BAMNET), a global consortium of researchers, breeders, and development organizations.^161^ BAMNET has played a crucial role in the genetic characterization, evaluation, and improvement of Bambara groundnut germplasm, leading to the development of enhanced yield potential, pod characteristics, and drought-tolerant, and nutritionally superior varieties making them more suitable for mechanized harvesting and processing.^162^ Commercialization efforts have focused on promoting its nutritious attributes in both local and international markets, contributing to enhanced food security and income generation for smallholder farmers.^163^

Furthermore, the promotion of Bambara groundnut’s nutritional profile, which includes high protein, fiber, and micronutrient content, has driven increased consumer demand and commercialization efforts. Local and regional value chains have been developed, providing smallholder farmers with better market access and income opportunities.^160^

### Cassava (Manihot Esculenta)

Cassava is widely cultivated for its starchy, tuberous roots, and to a lesser extent, leaves.^164^ It has been a basic food for smallholder farmers in sub-Saharan Africa since the 1950s. In the 1970s, breeding programs for cassava started, and cassava has moved from being a basic foodstuff to a cash crop.^165^ The improvement and commercialization of cassava can be attributed to the development and dissemination of improved varieties through initiatives like the Cassava: Adding Value for Africa (CAVA) project and the work of IITA, International Center for Tropical Agriculture (CIAT) and national agricultural research institutes like National Root Crops Research Institute (NRCRI).^166^ They developed the first wave of cassava mosaic virus disease (CMD) and cassava bacterial blight-resistant (CBB-resistant) Tropical Manihot Selection (TMS) varieties.^167^ Although conventional breeding was first used to improve cassava the development of improved varieties for disease resistance, enhanced nutritional value (provitamin A-rich cassava), improved yield, and stress tolerance (drought resistance) was sped up by the use of advanced biotechnology tools like genetic engineering, gene editing, and marker-assisted selection.^168^ Cassava, being a household staple food and the high demand for industrial starch products increased its awareness and commercialization.^169^ In the highest cassava-producing nation, Nigeria, cassava commercialization and market promotion programs were implemented between 2002 and 2008.^170^ This program, backed up with government policies saw to the development and distribution of cassava varieties resistant to cassava mosaic disease (CMD), promoting investment into cassava micro-processing and supporting small and large-scale processing industries. Value-added products such as High-Quality Cassava Flour (HQCF), starch, glucose syrup, and ethanol were produced by small and micro-scale processors increasing the production of cassava by 10 million tonnes in the six years of implementation.^165^

The genomic scientific community shifted its focus to cassava, and in 2009, the cassava reference genome was made public. Cassava was one of the first “orphan” crop genomes to be sequenced. Multiple cassava genome assemblies have been generated, and Single Nucleotide Polymorphism (SNP) has been used to generate a genetic map for cassava. Several transcriptomic data have also been generated and used for gene discovery, trait mapping, and genome editing.^171^ Advanced genomic tools like CRISPR/Cas9 have been completed to increase the yield of cassava, including disease resistance, herbicide tolerance, rapid flowering, and reduced cyanide content leaves and roots.^78^

### Sweet Potatoes (Ipomoea Batatas)

The journey of sweet potatoes from being an orphan crop to a staple crop is a remarkable success story, especially in regions like Africa and Asia. Initially regarded as a poor farmer’s crop, orange-fleshed sweet potatoes (OFSP) have gained recognition for their nutritional benefits, particularly in combating Vitamin A deficiency.^172^ While their strong flavor and high sweetness levels initially posed challenges to their popularity, concerted efforts in breeding, agronomy, and nutritional advocacy have played a significant role in overcoming these challenges.^173^ Breeding efforts focusing on enhancing β‐carotene, dry matter, and sugar content may have enhanced the transformation of sweet potatoes into a staple crop.^174^ Additionally, the development of value-added food products and the realization of sweet potatoes’ health benefits, such as low glycemic index and suitability for diabetics, have further solidified sweet potato’s position as a staple crop with significant importance and potential for diverse applications.^175^ Furthermore, efforts to increase awareness, investment in crop improvement, improve postharvest management, and expand market demand for sweet potato products have also been instrumental in promoting their adoption as a commercial crop.^176^

In terms of genomics, a genome sequencing initiative was officially launched in 2012. By 2015, the initial reference genome for the wild diploid sweet potato relative, *Ipomoea trifida*, was published, followed by the release of the sweet potato reference genome in 2017.^177^ Over the past few decades, traditional breeding and molecular breeding approaches have been widely applied to sweet potatoes and have resulted in the development of many new cultivars with improved yield, nutrient content, pest resistance, and other quality factors.^178^ Molecular breeding strategies, including marker-assisted selection and genomic selection, are being explored for sweet potato improvement. Numerous quantitative trait loci (QTLs) using biparental^179,180^ and GWAS^181,182^ have been identified for agronomic trait, quality traits, and biotic resistance in sweet potato Moreover, advanced genomic methods like CRISPR-Cas9 (clustered regularly interspaced short palindromic repeats-CRISPR-associated protein 9)-mediated mutagenesis via genome editing has also been used to develop improved varieties on starch quality.^183^ Methods like genomic selection (GS) have also been applied to accelerate genetic gains in sweet potatoes.^184^ New bioinformatics tools and pipelines have been developed to facilitate genetic and genomic research in the complex hexaploid sweet potato.^185^ The *Ipomoea batatas* genome browser, the Ipomoea Genome Hub, and the Sweet potato GARDEN are databases that provide access to the sweet potato genome, RNA-seq data, and other genomic resources.^177^

## Lesson learned from successful intervention

The successful transformation of some *orphan crops* into commercially viable and widely cultivated staples provides valuable lessons that can guide the future commercialization of other potential *orphan crops*.^186^ These lessons emphasize the importance of leveraging targeted research for consumer/framer preference, climate resilience, fostering collaborative efforts, developing markets, recognizing socioeconomic impacts, enacting supportive government policies, and integrating genomic research and breeding efforts.^187^ Climate resilience and nutritional value are critical factors in the success of *orphan crops*. Highlighting these attributes can significantly enhance the adoption and cultivation of such crops.^188^ For instance, crops with inherent drought resistance and high nutritional value have a better chance of being integrated into farming systems and diets. Promoting these benefits can drive consumer interest and farmer adoption, providing a strong foundation for further development and commercialization.

Targeted efforts on improving yield, disease resistance, and market appeal are crucial for the success of these crops.^189^ The use of advanced breeding techniques, such as marker-assisted selection, molecular breeding, and interspecific hybridization could be used to develop superior varieties that can overcome obstacles like pests, diseases, poor soil quality, and suboptimal agronomic practices, which often hinder the productivity and appeal of *orphan crops*. These advancements have accelerated breeding efforts and improved crop performance, making these crops more competitive in the market.^190^

Collaborative efforts among international and national research institutions for the development and improvement of orphan crops are instrumental. Such collaborations facilitate the sharing of knowledge, resources, and technologies, resulting in significant advancements in crop improvement.^191^ These partnerships are vital for conducting comprehensive research, developing superior germplasm, and ensuring that improved varieties reach farmers. Market development strategies are crucial for the commercial success of *orphan crops*. Developing market-specific traits, such as gluten-free properties or enhanced nutritional content, can open new commercial opportunities and increase the value of these crops.^18^ International recognition and marketing campaigns can elevate the status of these crops, facilitating their entry into global markets. Additionally, creating local and regional value chains can enhance market access and income opportunities for smallholder farmers.^92^ Supportive government policies and investments play a crucial role in the commercialization and market development of *orphan crops*. Government-backed programs that promote these crops as cash crops and support processing industries can significantly boost production and farmer incomes.^192^ Policies that encourage investment in agricultural research and development are also essential for the sustained growth of these crops.^188^

## Conclusions

The successful transformation of *orphan crops* into valuable staples points to the importance of targeted breeding programs, international collaboration, market development strategies, supportive government policies, and genomic research. Fast-tracking the improvement of orphan crops to improve desirable traits is more important than ever before. With the advent of new technologies and strategies, the hastening of orphan crop improvement should be possible and feasible. Modern technologies such as speed breeding and CRISPR/Cas9 in an integrative approach with genomic selection and other high-throughput methods could accelerate the breeding cycle of orphan crops. This will enable the development of new varieties with novel traits with more than one cultivation cycle per year. Still, with the various efforts being made worldwide, our expectation is that these crops will contribute immensely to the food and nutritional security of most households and may likely become staple crops for the world in the not-so-distant future.

## References

[1] Kumar B, Bhalothia P. Orphan crops for future food security. Journal of Biosciences. 2020;45(1). doi:10.1007/s12038-020-00107-5. 33184247

[2] Chapman MA, He Y, Zhou M. Beyond a reference genome: pangenomes and population genomics of underutilized and orphan crops for future food and nutrition security. New Phytologist. 2022;234(5):1583–97. doi:10.1111/NPH.18021. 35318683 PMC9994440

[3] Diouf D, Wang L, Hickey LT, Campbell BC. Editorial: harnessing resilience of orphan crops to the effects of drought and heat: a route towards a sustainable agriculture. Front Plant Sci. 2022:13. doi:10.3389/fpls.2022.1086143. PMC9780688 36570902

[4] Shahzad A, Ullah S, Dar AA, Sardar MF, Mehmood T, Tufail MA, Shakoor A, Haris M. Nexus on climate change: agriculture and possible solution to cope future climate change stresses. Environ Sci Pollut Res. 2021;28(12):14211–32. doi:10.1007/s11356-021-12649-8. 33515149

[5] Talabi AO, Vikram P, Thushar S, Rahman H, Ahmadzai H, Nhamo N, Shahid M, Singh RK. Orphan Crops : a Best Fit for Dietary Enrichment and Diversification in Highly Deteriorated Marginal Environments. Front Plant Sci. 2022;13:839704. doi: 10.3389/fpls.2022.839704 PMC8908242 35283935

[6] Tadele Z. Orphan crops: their importance and the urgency of improvement. Planta. 2019;250(3):677–94. doi:10.1007/S00425-019-03210-6. 31190115

[7] Mudau FN, Chimonyo VGP, Modi AT, Mabhaudhi T. Neglected and underutilised crops: a systematic review of their potential as food and herbal medicinal crops in South Africa. Front Pharmacol. 2022;12:809866. doi:10.3389/fphar.2021.809866. 35126143 PMC8811033

[8] Talabi AO, Vikram P, Thushar S, Rahman H, Ahmadzai H, Nhamo N, Shahid M, Singh RK. Orphan crops: a best fit for dietary enrichment and diversification in highly deteriorated marginal environments. Front Plant Sci. 2022;13:353. doi:10.3389/fpls.2022.839704. PMC8908242 35283935

[9] Diakostefani A, Velissaris R, Cvijanovic E, Bulgin R, Pantelides A, Leitch IJ, Mian S, Morton JA, Gomez MS, Chapman MA. Genome resources for underutilised legume crops: genome sizes, genome skimming and marker development. Genet Resour Crop Evol. 2023a:1–12. doi:10.1007/S10722-023-01636-2/FIGURES/3.

[10] Khoury CK, Brush S, Costich DE, Curry HA, de Haan S, Engels JMM, Guarino L, Hoban S, Mercer KL, Miller AJ, et al. Crop genetic erosion: understanding and responding to loss of crop diversity. New Phytologist. 2022;233(1):84–118. doi:10.1111/nph.17733. 34515358

[11] Dwivedi SL, Chapman MA, Abberton MT, Akpojotor UL, Ortiz R. Exploiting genetic and genomic resources to enhance productivity and abiotic stress adaptation of underutilized pulses. Frontiers in Genetics. 2023;14:1193780. doi:10.3389/fgene.2023.1193780. 37396035 PMC10311922

[12] Padulosi S, King EDIO, Hunter D, Swaminathan MS Orphan crops for sustainable food and nutrition security: promoting neglected and underutilized species. 1st ed. Routledge; 2021. doi:10.4324/9781003044802.

[13] Njaci I, Waweru B, Kamal N, Muktar MS, Fisher D, Gundlach H, Muli C, Muthui L, Maranga M, Kiambi D, et al. Chromosome-level genome assembly and population genomic resource to accelerate orphan crop lablab breeding. Nat Commun. 2023;14(1). doi:10.1038/s41467-023-37489-7. PMC10110558 37069152

[14] Vaijayanthi PV, Chandrakant, Ramesh S. Hyacinth bean (Lablab purpureus L. Sweet): genetics, breeding and genomics. Advances in Plant Breeding Strategies: Legumes. 2019;7:287–318. doi:10.1007/978-3-030-23400-3_8/COVER.

[15] Zhang G, Xu S, Mao W, Gong Y, Hu Q (2013). *Development of EST-SSR markers to study genetic diversity in hyacinth bean (Lablab purpureus L.)*. Plant OMICS. https://search.informit.org/doi/epdf/10.3316/informit.468435453446024

[16] Chapman MA. Transcriptome sequencing and marker development for four underutilized legumes. Applications in Plant Sciences. 2015;3(2):1400111. doi:10.3732/APPS.1400111. PMC4332146 25699221

[17] Maass BL, Chapman MA. The Lablab Genome: recent Advances and Future Perspectives. In: Chapman MA, editors. Underutilised Crop Genomes. Compendium of Plant Genomes. Cham: Springer; 2022. p. 229–53. doi:10.1007/978-3-031-00848-1_13.

[18] Gregory PJ, Mayes S, Hui CH, Jahanshiri E, Julkifle A, Kuppusamy G, Kuan HW, Lin TX, Massawe F, Suhairi TASTM, et al. Crops For the Future (CFF): an overview of research efforts in the adoption of underutilised species. Planta. 2019a;250(3):979–88. doi:10.1007/s00425-019-03179-2. 31250097

[19] Lepcha P, Egan AN, Doyle JJ, Sathyanarayana N. A review on current status and future prospects of winged bean (Psophocarpus tetragonolobus) in tropical agriculture. Plant Foods Human Nutr. 2017;72(3):225–35. doi:10.1007/s11130-017-0627-0. 28866817

[20] Sriwichai S, Monkham T, Sanitchon J, Jogloy S, Chankaew S. Dual-Purpose of the winged bean (Psophocarpus tetragonolobus (L.) DC.), the neglected tropical legume, based on pod and tuber yields. Plants. 2021;10(8):1746. doi:10.3390/plants10081746. 34451791 PMC8400048

[21] Vatanparast M, Shetty P, Chopra R, Doyle JJ, Sathyanarayana N, Egan AN. Transcriptome sequencing and marker development in winged bean (Psophocarpus tetragonolobus; Leguminosae). Sci Rep. 2016;6(1):1–14. doi:10.1038/srep29070. 27356763 PMC4928180

[22] Wong QN, Tanzi AS, Ho WK, Malla S, Blythe M, Karunaratne A, Massawe F, Mayes S. Development of gene-based SSR markers in winged bean (Psophocarpus tetragonolobus (L.) DC.) for diversity assessment. Genes. 2017;8(3):1–12. doi:10.3390/genes8030100.

[23] Chankaew S, Sriwichai S, Rakvong T, Monkham T, Sanitchon J, Tangphatsornruang S, Kongkachana W, Sonthirod C, Pootakham W, Amkul K, et al. The first genetic linkage map of winged bean [Psophocarpus tetragonolobus (L.) DC.] and QTL mapping for flower-, pod-, and seed-related traits. Plants. 2022;11(4):500. doi:10.3390/plants11040500. 35214834 PMC8878720

[24] Amkul K, Laosatit K, Lin Y, Yuan X, Chen X, Somta P. A gene encoding xylanase inhibitor is a candidate gene for. Plants. 2023;12(20):3602. doi:10.3390/plants12203602. 37896065 PMC10610162

[25] Dachapak S, Tomooka N, Somta P, Naito K, Kaga A, Srinives P, Papa R. QTL analysis of domestication syndrome in zombi pea (Vigna vexillata), an underutilized legume crop. PLoS ONE. 2018;13(12):1–22. doi:10.1371/journal.pone.0200116. PMC6298662 30562342

[26] Tripathi K, Gore PG, Pandey A, Nayar ER, Gayacharan C, Pamarthi RK, Bhardwaj R, Kumar A. Morphological and nutritional assessment of Vigna vexillata (L.) A. Rich.: a potential tuberous legume of India. Genet Resour Crop Evol. 2021;68(1):397–408. doi:10.1007/s10722-020-01023-1.

[27] Amkul K, Somta P, Laosatit K, Wang L. Identification of QTLs for domestication-related traits in zombi pea [Vigna vexillata (L.) A. Rich], a lost crop of Africa. Frontiers in Genetics. 2020;11(September):1–13. doi:10.3389/fgene.2020.00803. 33193562 PMC7530282

[28] Dachapak S, Somta P, Naito K, Tomooka N, Kaga A, Srinives P. Detection of quantitative trait loci for salt tolerance in zombi pea [Vigna vexillata (L.) A. Rich]. Euphytica. 2019;215(12):1–14. doi:10.1007/s10681-019-2530-2.

[29] Amkul K, Wang L, Somta P, Wang S, Cheng X. Construction of a high density linkage map and genome dissection of bruchid resistance in zombi pea (Vigna vexillata (L.) A. Rich). Sci Rep. 2019;9(1):1–10. doi:10.1038/s41598-019-48239-5. 31406222 PMC6690978

[30] Adewale BD, Vroh-Bi I, Dumet DJ, Nnadi S, Kehinde OB, Ojo DK, Adegbite AE, Franco J. Genetic diversity in African yam bean accessions based on AFLP markers: towards a platform for germplasm improvement and utilization. Plant Genetic Res Charact Utilisation. 2015;13(2):111–18. doi:10.1017/S1479262114000707.

[31] Raji M, Akinosun A, Makanju T, Oluwemimo B. Potentials of toasted African yam bean seed meal supplemented with multi-enzyme on performance characteristics and blood serum of broiler starter chickens. Nigerian J. Anim. Sci. 2019;21:64–70.

[32] Nnamani CV, Ajayi SA, Oselebe HO, Atkinson CJ, Igboabuchi AN, Ezigbo EC. Sphenostylis stenocarpa (Ex. A. Rich.) harms. a fading genetic resource in a changing climate: prerequisite for conservation and sustainability. Plants. 2017;6(3):30. doi:10.3390/plants6030030. 28704944 PMC5620586

[33] Arisa NU, Ogbuele OC. Production, quality assessment and acceptability of African yam bean Sphenostylis stenoscarpa sauce. J Food Process Preserv. 2007;31(6):771–78. doi:10.1111/j.1745-4549.2007.00152.x.

[34] Klu GYP, Amoatey HM, Bansa D, Kumaga FK. Cultivation and use of African yam bean (*Sphenostylis stenocarpa*) in the volta region of Ghana. J Food Technol Africa. 2001;6(3):1–9. doi:10.4314/jfta.v6i3.19292.

[35] Gbenga-Fabusiwa FJ. African yam beans (Sphenostylis stenocarpa): a review of a novel tropical food plant for human nutrition, health and food security. Afr J Food Sci 2021;15(2):33–47. doi:10.5897/ajfs2020.1961.

[36] Olomitutu OE, Paliwal R, Abe A, Oluwole OO, Oyatomi OA, Abberton MT. Genome-wide association study revealed SNP alleles associated with seed size traits in African Yam Bean (Sphenostylis stenocarpa (Hochst ex. A. Rich.) Harms). Genes. 2022;13(12):2350. doi:10.3390/genes13122350. 36553617 PMC9777823

[37] Oluwole OO, Olomitutu OE, Paliwal R, Oyatomi OA, Abberton MT, Obembe OO. Preliminary assessment of the association between dart-seq snp some nutritional traits in African yam bean. Tropical Journal of Natural Product Research. 2020;4(11):877–79. doi:10.26538/tjnpr/v4i11.5.

[38] Adjei RR, Donkor EF, Santo KG, Adarkwah C, Sarfo Boateng A, Afreh DN, et al. A preliminary evaluation of variability, genetic estimates, and association among phenotypic traits of African yam bean landraces from Ghana. Adv Agric. 2023;2023(1):1996255.

[39] Shitta NS, Unachukwu N, Edemodu AC, Abebe AT, Oselebe HO, Abtew WG. Genetic diversity and population structure of an African yam bean (Sphenostylis stenocarpa) collection from IITA GenBank. Sci Rep. 2022;12(1):1–13. doi:10.1038/s41598-022-08271-4. 35292678 PMC8924269

[40] Dada AO, Oresanya A, Akinyosoye ST, Arogundade O. The first report of bean common mosaic virus (BCMV) infection of African yam bean (*Sphenostylis stenocarpa*) in Nigeria. Mol Biol Rep. 2022;49(10):10133–36. 36028731 10.1007/s11033-022-07883-3

[41] Akinyosoye S. Characterization of African Yam Bean (*Sphenostylis stenocarpa*) mutant lines using phenotypic markers. Yuz Yıl Univ J Agric Sci. 2022;32(3):487–96.

[42] Akinyosoye ST, Adetumbi JA, Ukachukwu PC. Impact of sodium azide on maturity, seed and tuber yields in M1 african yam bean [*Sphenostylis stenocarpa* (Hochst Ex A. rich) harms] generation. Trop Agric. 2021;98(1):16–27.

[43] Olawuyi OJ, Inyang VE, Oladele DD, David SC. Mitotic studies, pollen fertility and morphological response of African yam bean (*Sphenostylis stenocarpa* (Hochst. ex A. Rich) Harms) to gamma radiation. J Crop Sci Biotechnol. 2023;26(4):499–510.

[44] Chang Y, Liu H, Liu M, Liao X, Sahu SK, Fu Y, Song B, Cheng S, Kariba R, Muthemba S, et al. The draft genomes of five agriculturally important African orphan crops. GigaScience. 2019;8(3):1–16. doi:10.1093/GIGASCIENCE/GIY152. PMC6405277 30535374

[45] Naeem M, Shabbir A, Ansari AA, Aftab T, Khan MMA, Uddin M. Hyacinth bean (Lablab purpureus L.) – an underutilised crop with future potential. Scientia Horticulturae. 2020;272:109551. doi:10.1016/J.SCIENTA.2020.109551.

[46] Butsayawarapat P, Juntawong P, Khamsuk O, Somta P. Comparative transcriptome analysis of waterlogging-sensitive and tolerant Zombi pea (Vigna vexillata) reveals energy conservation and root plasticity controlling waterlogging tolerance. Plants. 2019;8(8):264. doi:10.3390/plants8080264. 31382508 PMC6724125

[47] Diakostefani A, Velissaris R, Cvijanovic E, Bulgin R, Pantelides A, Leitch IJ, Mian S, Morton JA, Gomez MS, Chapman MA. Genome resources for underutilised legume crops: genome sizes, genome skimming and marker development. Genet Resour Crop Evol. 2023:0123456789. doi:10.1007/s10722-023-01636-2.

[48] Oluwole OO, Aworunse OS, Aina AI, Oyesola OL, Popoola JO, Oyatomi OA, Abberton MT, Obembe OO. A review of biotechnological approaches towards crop improvement in African yam bean (Sphenostylis stenocarpa Hochst. Ex A. Rich.). Heliyon. 2021;7(11):e08481. doi:10.1016/j.heliyon.2021.e08481. 34901510 PMC8642607

[49] Bhadmus A, Abberton M, Idehen E, Ekanem U, Paliwal R, Oyatomi O. Genetic diversity assessment of winged bean [Psophocarpus tetragonolobus (L.) DC.] accessions using agronomic and seed morphometric traits. Crops. 2023;3(2):170–83. doi:10.3390/crops3020017.

[50] Chelangat M, Muturi P, Gichimu B, Gitari J, Mukono S, Addi M. Nutritional and phytochemical composition of bambara groundnut (Vigna subterranea [L.] Verdc) Landraces in Kenya. Int J Agronomy. 2023;2023:1–11. doi:10.1155/2023/9881028.

[51] Veldsman Z, Pretorius B, Schönfeldt HC. Examining the contribution of an underutilized food source, Bambara Groundnut, in improving protein intake in Sub-Saharan Africa. Frontiers in Sustainable Food Systems. 2023;7(August). doi:10.3389/fsufs.2023.1183890.

[52] Hatakeyama M, Aluri S, Thumilan Balachadran M, Sivarajan SR, Patrignani A, Grü Ter S, Poveda L, Shimizu-Inatsugi R, Baeten J, Francoijs K-J, et al. Multiple hybrid de novo genome assembly of finger millet, an orphan allotetraploid crop. Dna Research. 2017;0:1–9. doi:10.1093/dnares/dsx036. PMC5824816 28985356

[53] Sood S, Joshi DC, Chandra AK, Kumar A. Phenomics and genomics of finger millet: current status and future prospects. Planta. 2019;250(3):731–51. doi:10.1007/S00425-019-03159-6. 30968267

[54] Arriagada O, Cacciuttolo F, Cabeza RA, Carrasco B, Schwember AR. A comprehensive review on chickpea (Cicer arietinum L.) breeding for abiotic stress tolerance and climate change resilience. Int J Mol Sci. 2022;23(12):6794. doi:10.3390/IJMS23126794. 35743237 PMC9223724

[55] Jain M, Misra G, Patel RK, Priya P, Jhanwar S, Khan AW, Shah N, Singh VK, Garg R, Jeena G, et al. A draft genome sequence of the pulse crop chickpea (Cicer arietinum L.). The Plant Journal. 2013;74(5):715–29. doi:10.1111/TPJ.12173. 23489434

[56] Wang J, Zhang G, Liu X, Quan Z, Cheng S, Xu X, Pan S, Xie M, Zeng P, Yue Z, et al. Genome sequence of foxtail millet (Setaria italica) provides insights into grass evolution and biofuel potential. Nature Biotechnol. 2012;30(6):549–54. doi:10.1038/nbt.2195. 22580950

[57] Liu T, Yang X, Batchelor WD, Liu Z, Zhang Z, Wan N, Sun S, He B, Gao J, Bai F, et al. A case study of climate-smart management in foxtail millet (Setaria italica) production under future climate change in Lishu county of Jilin, China. Agricultural and Forest Meteorology. 2020;292-293:108131. doi:10.1016/J.AGRFORMET.2020.108131.

[58] Emmrich PMF, Sarkar A, Njaci I, Kaithakottil GG, Ellis N, Moore C, Edwards A, Heavens D, Waite D, Cheema J, et al. A draft genome of grass pea (Lathyrus sativus), a resilient diploid legume. BioRxiv. 2020;4(24):058164. doi:10.1101/2020.04.24.058164

[59] Rathi D, Chakraborty S, Chakraborty N. Grasspea, a critical recruit among neglected and underutilized legumes, for tapping genomic resources. Current Plant Biology. 2021;26:100200. doi:10.1016/J.CPB.2021.100200.

[60] Bhardwaj J, Chauhan R, Swarnkar MK, Chahota RK, Singh AK, Shankar R, Yadav SK. Comprehensive transcriptomic study on horse gram (Macrotyloma uniflorum): de novo assembly, functional characterization and comparative analysis in relation to drought stress. BMC Genomics. 2013;14(1):1–18. doi:10.1186/1471-2164-14-647. 24059455 PMC3853109

[61] Jha UC, Nayyar H, Parida SK, Siddique KHM. Horse gram, an underutilized climate-resilient legume: breeding and genomic approach for improving future genetic gain. Developing Climate Resilient Grain Forage Legumes. 2022:167–78. doi:10.1007/978-981-16-9848-4_8/FIGURES/1.

[62] Varshney RK, Chen W, Li Y, Bharti AK, Saxena RK, Schlueter JA, Donoghue MTA, Azam S, Fan G, Whaley AM, et al. Draft genome sequence of pigeonpea (Cajanus cajan), an orphan legume crop of resource-poor farmers. Nature Biotechnol. 2011;30(1). doi:10.1038/nbt.2022. 22057054

[63] Saxena KB, Sultana R, Bhatnagar-Mathur P, Saxena RK, Chauhan YS, Kumar RV, Singh IP, Raje RS, Tikle AN. Accomplishments and challenges of pigeonpea breeding research in India. Indian Journal of Genetics and Plant Breeding. 2016;76(4):467–82. doi:10.5958/0975-6906.2016.00065.1.

[64] Rani J, Dhull SB, Kinabo J, Kidwai MK, Sangwan A. A narrative review on nutritional and health benefits of underutilized summer crop to address agriculture challenges: moth bean (Vigna aconitifoliaL.). Legume Science. 2023;5(4):e204. doi:10.1002/LEG3.204.

[65] Suranjika S, Pradhan S, Kalia RK, Dey N. De novo assembly of the whole genome of Moth bean (Vigna aconitifolia), an underutilized Vigna species of India. BioRxiv. 2023;5(18):540937. doi:10.1101/2023.05.18.540937

[66] Jegadeesan S, Raizada A, Dhanasekar P, Suprasanna P. Draft genome sequence of the pulse crop blackgram [Vigna mungo (L.) Hepper] reveals potential R-genes. Scientific Reports 2021. 2021;11(1):1–10. doi:10.1038/s41598-021-90683-9. PMC8160138 34045617

[67] Prajapati PK, Singh AP. Investgation of molecular variability on MYMV resistant and susceptible blackgram specie using SSR marker. Int J Res Anal Rev. 2022;9(1):192–215. http://www.ijrar.org/papers/IJRAR22A1028.pdf.

[68] Kang YJ, Satyawan D, Shim S, Lee T, Lee J, Hwang WJ, Kim SK, Lestari P, Laosatit K, Kim KH, et al. Draft genome sequence of adzuki bean, Vigna angularis. Sci Rep. 2015;5(1):1–8. doi:10.1038/srep08069. PMC5389050 25626881

[69] Li H, Zou L, Li XY, Wu DT, Liu HY, Li HB, Gan RY. Adzuki bean (Vigna angularis): chemical compositions, physicochemical properties, health benefits, and food applications. Comprehensive Reviews in Food Science and Food Safety. 2022;21(3):2335–62. doi:10.1111/1541-4337.12945. 35365946

[70] Zhao Q, Hou D, Laraib Y, Xue Y, Shen Q. Comparison of the effects of raw and cooked adzuki bean on glucose/lipid metabolism and liver function in diabetic mice. Cereal Chem. 2021;98(5):1081–90. doi:10.1002/CCHE.10456.

[71] Chen H, Wang L, Wang S, Liu C, Blair MW, Cheng X, Jain M. Transcriptome sequencing of mung bean (Vigna radiate L.) genes and the identification of EST-SSR markers. PLOS ONE. 2015;10(4):e0120273. doi:10.1371/JOURNAL.PONE.0120273. 25830701 PMC4382333

[72] Hou D, Yousaf L, Xue Y, Hu J, Wu J, Hu X, Feng N, Shen Q. Mung bean (Vigna radiata L.): bioactive polyphenols, polysaccharides, peptides, and health benefits. Nutrients. 2019;11(6):1238. doi:10.3390/NU11061238. 31159173 PMC6627095

[73] Wang X, Chen S, Ma X, Yssel AEJ, Chaluvadi SR, Johnson MS, Gangashetty P, Hamidou F, Sanogo MD, Zwaenepoel A, et al. Genome sequence and genetic diversity analysis of an under-domesticated orphan crop, white fonio (Digitaria exilis). GigaScience. 2021a;10(3):1–12. doi:10.1093/GIGASCIENCE/GIAB013. PMC7953496 33710327

[74] Popoola JO, Aworunse OS, Ojuederie OB, Adewale BD, Ajani OC, Oyatomi OA, Eruemulor DI, Adegboyega TT, Obembe OO. The exploitation of orphan legumes for food, income, and nutrition security in Sub-Saharan Africa. Front Plant Sci. 2022;13:782140. doi:10.3389/fpls.2022.782140. 35665143 PMC9156806

[75] Jangra S, Chaudhary V, Yadav RC, Yadav NR. High-throughput phenotyping: a platform to accelerate crop improvement. Phenomics. 2021;1(2):31–53. doi:10.1007/S43657-020-00007-6. 36939738 PMC9590473

[76] Mohammadi R, Golkari S. Genetic resources for enhancing drought tolerance from a mini-core collection of spring bread wheat (*Triticum aestivum* L.). Acta Scientiarum. Agronomy. 2022;44:e56129. doi:10.4025/ACTASCIAGRON.V44I1.56129.

[77] Upadhyaya HD, Dronavalli N, Gowda CLL, Singh S (2012). *Mini Core Collections for Enhanced Utilization of Genetic Resources in Crop Improvement*. http://www.indianjournals.com/ijor.aspx?target=ijor:ijpgr&volume=25&issue=1&article=010

[78] Yaqoob H, Tariq A, Bhat BA, Bhat KA, Nehvi IB, Raza A, Djalovic I, Prasad PV, Mir RA. Integrating genomics and genome editing for orphan crop improvement: a bridge between orphan crops and modern agriculture system. GM Crops Food. 2023;14(1):1–20. doi:10.1080/21645698.2022.2146952. PMC9828793 36606637

[79] Movahedi A, Pucker B, Kadkhodaei S. Editorial: genomics and gene editing of orphan plants. Front Plant Sci. 2023;14(September). doi:10.3389/fpls.2023.1277625. PMC10534993 37780490

[80] Rebetzke GJ, Jimenez-Berni J, Fischer RA, Deery DM, Smith DJ. Review: high-throughput phenotyping to enhance the use of crop genetic resources. Plant Science. 2019;282:40–48. doi:10.1016/J.PLANTSCI.2018.06.017. 31003610

[81] Kamenya SN, Mikwa EO, Song B, Odeny DA. Genetics and breeding for climate change in Orphan crops. Theoretical and Applied Genetics. 2021;134(6):1787–815. doi:10.1007/S00122-020-03755-1. 33486565 PMC8205878

[82] Succurro A, Schuler-Bermann M, Ivanov R, Jacoby R, Kopriva S, Jobe TO. Orphan crops at the food for future conference. Planta. 2019;250(3):1005–10. doi:10.1007/s00425-019-03229-9. 31290030

[83] Ibrahim Bio Yerima AR, Achigan-Dako EG. A review of the orphan small grain cereals improvement with a comprehensive plan for genomics-assisted breeding of fonio millet in West Africa. Plant Breed. 2021;140(4):561–74. doi:10.1111/PBR.12930.

[84] Großkinsky DK, Faure JD, Gibon Y, Haslam RP, Usadel B, Zanetti F, Jonak C. The potential of integrative phenomics to harness underutilized crops for improving stress resilience. Front Plant Sci. 2023;14:1216337. doi:10.3389/fpls.2023.1216337. 37409292 PMC10318926

[85] Zhang A, Liu Y, Wang F, Li T, Chen Z, Kong D, Bi J, Zhang F, Luo X, Wang J, et al. Enhanced rice salinity tolerance via CRISPR/Cas9-targeted mutagenesis of the OsRR22 gene. Mol Breed. 2019a;39(3):1–10. doi:10.1007/s11032-019-0954-y. PMC7413041 32803201

[86] Dissanayake R, Kahrood HV, Dimech AM, Noy DM, Rosewarne GM, Smith KF, Cogan NOI, Kaur S. Development and application of image-based high-throughput phenotyping methodology for salt tolerance in lentils. Agronomy. 2020;10(12):1992. doi:10.3390/AGRONOMY10121992.

[87] Madurapperumage A, Johnson N, Thavarajah P, Tang L, Thavarajah D. Fourier-transform infrared spectroscopy (FTIR) as a high-throughput phenotyping tool for quantifying protein quality in pulse crops. The Plant Phenome Journal. 2022;5(1):e20047. doi:10.1002/PPJ2.20047.

[88] Afanasenko O, Rozanova I, Gofman A, Lashina N, Novakazi F, Mironenko N, Baranova O, Zubkovich A. Validation of molecular markers of barley net blotch resistance loci on chromosome 3H for marker-assisted selection. Agriculture (Switzerland). 2022;12(4):439. doi:10.3390/AGRICULTURE12040439/S1.

[89] Olasanmi B, Kyallo M, Yao N. Marker-assisted selection complements phenotypic screening at seedling stage to identify cassava mosaic disease-resistant genotypes in African cassava populations. Sci Rep. 2021;11(1):1–8. doi:10.1038/s41598-021-82360-8. 33531574 PMC7854622

[90] Rani A, Taunk J, Jangra S, Yadav RC, Yadav NR, Yadav D, Yadav HP. Development of advance pearl millet lines tolerant to terminal drought stress using marker-assisted selection. Vegetos. 2022;35(1):63–73. doi:10.1007/s42535-021-00284-0.

[91] Tang Y, Qiu X, Hu C, Li J, Wu L, Wang W, Li X, Li X, Zhu H, Sui J, et al. Breeding of a new variety of peanut with high-oleic-acid content and high-yield by marker-assisted backcrossing. Mol Breed. 2022;42(7):1–15. doi:10.1007/s11032-022-01313-9. 37313504 PMC10248636

[92] Singh AK, Singh S, Saroj PL, Mishra DS, Yadav V, Kumar R. Cultivation of underutilized fruit crops in hot semi-arid regions: developments and challenges — a review. Current Horticulture. 2020;8(1):12. doi:10.5958/2455-7560.2020.00003.5.

[93] Cheng A, Chai HH, Ho WK, Bamba ASA, Feldman A, Kendabie P, Halim RA, Tanzi A, Mayes S, Massawe F. Molecular marker technology for genetic improvement of underutilised crops. Crop Improvement. 2017:47–70. doi:10.1007/978-3-319-65079-1_3.

[94] Zhang G, Liu X, Quan Z, Cheng S, Xu X, Pan S, Xie M, Zeng P, Yue Z, Wang W, et al. Genome sequence of foxtail millet (Setaria italica) provides insights into grass evolution and biofuel potential. Nature Biotechnol. 2012;30(6):549–54. doi:10.1038/nbt.2195. 22580950

[95] Chanyalew S, Ferede S, Damte T, Fikre T, Genet Y, Kebede W, Tolossa K, Tadele Z, Assefa K. Significance and prospects of an orphan crop tef. Planta. 2019;250(3):753–67. doi:10.1007/s00425-019-03209-z. 31222492

[96] Barkley NA, Wang ML, Gillaspie AG, Dean RE, Pederson GA, Jenkins TM. Discovering and verifying DNA polymorphisms in a mung bean [V. radiata(L.) R. Wilczek] collection by EcoTILLING and sequencing. BMC Research Notes. 2008;1(1):1–7. doi:10.1186/1756-0500-1-28. 18710546 PMC2518284

[97] Bajaj D, Srivastava R, Nath M, Tripathi S, Bharadwaj C, Upadhyaya HD, Tyagi AK, Parida SK. Eco TILLING-based association mapping efficiently delineates functionally relevant natural allelic variants of candidate genes governing agronomic traits in chickpea. Front Plant Sci. 2016;7(APR2016):179815. doi:10.3389/fpls.2016.00450. PMC4835497 27148286

[98] Uba CU, Oselebe HO, Tesfaye AA, Abtew WG. Association mapping in bambara groundnut [Vigna subterranea (L.) Verdc.] reveals loci associated with agro-morphological traits. BMC Genomics. 2023;24(1):1–14. doi:10.1186/s12864-023-09684-9. 37803263 PMC10557193

[99] Reddy VRP, Das S, Dikshit HK, Mishra GP, Aski MS, Singh A, Tripathi K, Pandey R, Bansal R, Singh MP, et al. Genetic dissection of phosphorous uptake and utilization efficiency traits using gwas in mungbean. Agronomy. 2021;11(7):1401. doi:10.3390/agronomy11071401.

[100] Xu N, Chen B, Cheng Y, Su Y, Song M, Guo R, Wang M, Deng K, Lan T, Bao S, et al. Integration of GWAS and RNA-seq analysis to identify SNPs and candidate genes associated with alkali stress tolerance at the germination stage in mung bean. Genes. 2023;14(6):1294. doi:10.3390/genes14061294. 37372474 PMC10298294

[101] Yadav AK, Singh CK, Kalia RK, Mittal S, Wankhede DP, Kakani RK, Ujjainwal S, Aakash, Saroha A, Nathawat NS, et al. Genetic diversity, population structure, and genome-wide association study for the flowering trait in a diverse panel of 428 moth bean (Vigna aconitifolia) accessions using genotyping by sequencing. BMC Plant Biol. 2023;23(1):1–17. doi:10.1186/s12870-023-04215-w. 37120525 PMC10148550

[102] Singh RK, Prasad A, Muthamilarasan M, Parida SK, Prasad M. Breeding and biotechnological interventions for trait improvement: status and prospects. Planta. 2020b;252(4):1–18. doi:10.1007/s00425-020-03465-4. 32948920 PMC7500504

[103] Watson A, Ghosh S, Williams MJ, Cuddy WS, Simmonds J, Rey MD, Asyraf Md Hatta M, Hinchliffe A, Steed A, Reynolds D, et al. Speed breeding is a powerful tool to accelerate crop research and breeding. Nature Plants. 2018;4(1):23–29. doi:10.1038/s41477-017-0083-8. 29292376

[104] Samineni S, Sen M, Sajja SB, Gaur PM. Rapid generation advance (RGA) in chickpea to produce up to seven generations per year and enable speed breeding. Crop J. 2020;8(1):164–69. doi:10.1016/J.CJ.2019.08.003.

[105] Michno JM, Wang X, Liu J, Curtin SJ, Kono TJ, Stupar RM. CRISPR/Cas mutagenesis of soybean and Medicago truncatula using a new web-tool and a modified Cas9 enzyme. GM Crops Food. 2015;6(4):243–52. doi:10.1080/21645698.2015.1106063. 26479970 PMC5033229

[106] Ji J, Zhang C, Sun Z, Wang L, Duanmu D, Fan Q. Genome editing in cowpea vigna unguiculata using CRISPR-Cas9. Int J Mol Sci. 2019;20(10):2471. doi:10.3390/IJMS20102471. 31109137 PMC6566367

[107] Zhang C, Chen W, Sankaran S. High-throughput field phenotyping of Ascochyta blight disease severity in chickpea. Crop Protection. 2019;125:104885. doi:10.1016/J.CROPRO.2019.104885.

[108] Juma BS, Mukami A, Mweu C, Ngugi MP, Mbinda W. Targeted mutagenesis of the CYP79D1 gene via CRISPR/Cas9-mediated genome editing results in lower levels of cyanide in cassava. Front Plant Sci. 2022;13:1009860. doi:10.3389/fpls.2022.1009860. 36388608 PMC9644188

[109] Ahmar S, Gruszka D. CRISPR/Cas9 boosts wheat yield by reducing brassinosteroid signaling. Trends Biochem Sci. 2023;48(11):917–19. doi:10.1016/j.tibs.2023.07.005. 37517884

[110] Lemmon ZH, Reem NT, Dalrymple J, Soyk S, Swartwood KE, Rodriguez-Leal D, Van Eck J, Lippman ZB. Rapid improvement of domestication traits in an orphan crop by genome editing. Nature Plants. 2018;4(10):766–70. doi:10.1038/S41477-018-0259-X. 30287957

[111] Ye Q, Meng X, Chen H, Wu J, Zheng L, Shen C, Guo D, Zhao Y, Liu J, Xue Q, et al. Construction of genic male sterility system by CRISPR/Cas9 editing from model legume to alfalfa. Plant Biotechnology Journal. 2022;20(4):613. doi:10.1111/PBI.13770. 34962045 PMC8989503

[112] Lin CS, Hsu CT, Yang LH, Lee LY, Fu JY, Cheng QW, Wu FH, Hsiao HCW, Zhang Y, Zhang R, et al. Application of protoplast technology to CRISPR/Cas9 mutagenesis: from single-cell mutation detection to mutant plant regeneration. Plant Biotechnology Journal. 2018;16(7):1295–310. doi:10.1111/PBI.12870. 29230929 PMC5999315

[113] Duan K, Cheng Y, Ji J, Wang C, Wei Y, Wang Y. Large chromosomal segment deletions by CRISPR/LbCpf1-mediated multiplex gene editing in soybean. Journal of Integrative Plant Biology. 2021;63(9):1620–31. doi:10.1111/jipb.13158. 34331750

[114] Liu X, Zhang S, Jiang Y, Yan T, Fang C, Hou Q, Wu S, Xie K, An X, Wan X. Use of CRISPR/Cas9-based gene editing to simultaneously mutate multiple homologous genes required for pollen development and male fertility in maize. Cells. 2022;11(3):439. doi:10.3390/cells11030439. 35159251 PMC8834288

[115] Maioli A, Gianoglio S, Moglia A, Acquadro A, Valentino D, Milani AM, Prohens J, Orzaez D, Granell A, Lanteri S, et al. Simultaneous CRISPR/Cas9 editing of three PPO genes reduces fruit flesh browning in solanum melongena L. Front Plant Sci. 2020;11:607161. doi:10.3389/fpls.2020.607161. 33343607 PMC7744776

[116] Liang Z, Wu Y, Ma L, Guo Y, Ran Y. Efficient genome editing in setaria italica using CRISPR/Cas9 and base editors. Front Plant Sci. 2022;12:815946. doi:10.3389/fpls.2021.815946. 35095986 PMC8793480

[117] Badhan S, Ball AS, Mantri N. First report of CRISPR/Cas9 mediated DNA-free editing of 4CL and RVE7 genes in chickpea protoplasts. Int J Mol Sci. 2021;22(1):396. doi:10.3390/IJMS22010396. 33401455 PMC7795094

[118] Venezia M, Creasey Krainer KM. Current advancements and limitations of gene editing in orphan crops. Front Plant Sci. 2021;12:742932. doi:10.3389/fpls.2021.742932. 34630494 PMC8493294

[119] AOCC. African Orphan Crops Consortium – Healthy Africa through nutritious, diverse and local food crops [Internet]. 2024 [cited Nov 17]. Available from https://africanorphancrops.org/.

[120] Opole RA. Opportunities for enhancing production, utilization and marketing of Finger Millet in Africa. Afr J Food Agric Nutr Dev. 2019;19(1):13863–82. doi:10.18697/AJFAND.84.BLFB1004.

[121] McMullin S, Stadlmayr B, Mausch K, Revoredo-Giha C, Burnett F, Guarino L, Brouwer ID, Jamnadass R, Graudal L, Powell W, et al. Determining appropriate interventions to mainstream nutritious orphan crops into African food systems. Global Food Security. 2021;28:100465. doi:10.1016/J.GFS.2020.100465.

[122] Padulosi S, Amaya K, Jäger M, Gotor E, Rojas W, Valdivia R. A holistic approach to enhance the use of neglected and underutilized species: the case of andean grains in bolivia and peru. Sustainability. 2014;6(3):1283–312. doi:10.3390/SU6031283.

[123] Camacho-Henriquez A, Kraemer F, Galluzzi G, de Haan S, Jäger M, Christinck A. Decentralized collaborative plant breeding for utilization and conservation of neglected and underutilized crop genetic resources. Advances in Plant Breeding Strategies: Breeding, Biotechnology and Molecular Tools. 2015;1:25–61. doi:10.1007/978-3-319-22521-0_2.

[124] Putsenteilo P, Klapkiv Y, Karpenko V, Gvozdecka I. The role of institutions in the development of agriculture. Bulgarian Journal of Agricultural Science. 2020;26:23–33.

[125] Thind TS, Dhillon BS. Sustainable development of agriculture for national food and nutritional security and farmers’ livelihood. Agricultural Research Journal. 2021;58(3):545. doi:10.5958/2395-146X.2021.00080.6.

[126] Borelli T, Hunter D, Padulosi S, Amaya N, Meldrum G, de Oliveira Beltrame DM, Samarasinghe G, Wasike VW, Güner B, Tan A, et al. Local solutions for sustainable food systems: the contribution of orphan crops and wild edible species. Agronomy. 2020;10(2):231. doi:10.3390/AGRONOMY10020231.

[127] Ribaut J-M, Ragot M. Modernising breeding for orphan crops: tools, methodologies, and beyond. Planta. 2019;250(3):971–77. doi:10.1007/s00425-019-03200-8. 31256257

[128] Ceccarelli S, Grando S. Participatory plant breeding: who did it, who does it and where? Experimental Agriculture. 2020;56(1):1–11. doi:10.1017/S0014479719000127.

[129] Chirwa R (2017). *Variety Development Strategy and Stage Plan*. https://cabidigitallibrary.org

[130] Caetano CM, Peña CRD, Maigual JJL, Vásquez DLN, Nunes DC, Pazdiora BRCN. participatory breeding: tool for conservation of neglected and underutilized crops. Rapid Communications in Mass Spectrometry: Rcm. 2015;64(3sup):307–27. doi:10.15446/ACAG.V64N3SUP.50550.

[131] Ndinya C, Onyango E, Dinssa F, Odendo M, Simon J. Participatory variety selection of three African leafy vegetables in Western Kenya. Journal of Medicinally Active Plants. 2020; 9(3): 145. https://scholarworks.umass.edu/jmap/vol9/iss3/6

[132] Colley MR, Dawson JC, McCluskey C, Myers JR, Tracy WF, Lammerts Van Bueren ET. Exploring the emergence of participatory plant breeding in countries of the Global North – a review. The Journal of Agricultural Science. 2021;159(5–6):320–38. doi:10.1017/S0021859621000782.

[133] Almekinders CJM, Mertens L, van Loon JP, Lammerts van Bueren ET. Potato breeding in the Netherlands: a successful participatory model with collaboration between farmers and commercial breeders. Food Security. 2014;6(4):515–24. doi:10.1007/s12571-014-0369-x.

[134] Choudhary AK, Kumar S, Kumar S Breeding cultivars for intensifying rice-fallow cropping system. 2023.

[135] Commission on genetic resources for food and agriculture - FAO. (2010). The Second Report on THE STATE OF THE WORLD ’ s PLANT GENETIC RESOURCES FOR The Second Report on THE STATE OF THE WORLD ’ s PLANT GENETIC RESOURCES FOR. In *Organization* (Issue January).

[136] Mascher M, Schreiber M, Scholz U, Graner A, Reif JC, Stein N. Genebank genomics bridges the gap between the conservation of crop diversity and plant breeding. Nature Genetics. 2019;51(7):1076–81. doi:10.1038/s41588-019-0443-6. 31253974

[137] Wambugu PW, Ndjiondjop MN, Henry RJ. Role of genomics in promoting the utilization of plant genetic resources in genebanks. Briefings in Functional Genomics. 2018;17(3):198–206. doi:10.1093/BFGP/ELY014. 29688255 PMC5967547

[138] Anumalla M, Roychowdhury R, Kumar Geda C, Mazid M, Rathoure AK. Utilization of plant genetic resources and diversity analysis tools for sustainable crop improvement with special emphasis on rice. Int J Adv Res. 2015; 3(3): 1155–75. http://www.journalijar.com

[139] Kamenya SN, Mikwa EO, Song B, Odeny DA. Genetics and breeding for climate change in orphan crops. Theor Appl Genet. 2021 [cited 2023 Oct 26]; 134(6):1787–815. https://link.springer.com/article/10.1007/s00122-020-03755-1. 33486565 10.1007/s00122-020-03755-1 PMC8205878

[140] Joshi P, Gupta SK, Ojulong H, Sharma R, Vetriventhan M, Kudapa H, Choudhary S, Naresh D, Kholova J, Sajja S. Finger millet improvement in post-genomic era: hundred years of breeding and moving forward. Smart Plant Breeding Field Crops Post-Genomics Era. 2023:221–53. doi:10.1007/978-981-19-8218-7_7.

[141] Gebreyohannes A, Shimelis H, Mashilo J, Odeny DA, Tadesse T, Ojiewo CO. Finger millet (Eleusine coracana) improvement: challenges and prospects—A review. Plant Breed. 2024;143(3):350–74. doi:10.1111/PBR.13169.

[142] Kumar A, Metwal M, Kaur S, Gupta AK, Puranik S, Singh S, Singh M, Gupta S, Babu BK, Sood S, et al. Nutraceutical value of finger millet [Eleusine coracana (L.) Gaertn.], and their improvement using omics approaches. Front Plant Sci. 2016;7:198656. doi:10.3389/fpls.2016.00934. PMC4925701 27446162

[143] Mirza N, Marla SS. Finger Millet (Eleusine coracana L. Gartn.) Breeding. Adv Plant Breed Strategies:. 2019;5:83–132. doi:10.1007/978-3-030-23108-8_3.

[144] Stories IS. ICRISAT success stories. 2014;6.

[145] Mahesh HB, Manasa KG, Raghavendra NR, Shirke MD, Hittalmani S The Complete Genome Sequence of Finger Millet. In: Kumar A, Sood S, Babu BK, Gupta SM, Rao BD, editors. The Finger Millet Genome. Compendium of Plant Genomes. Cham: Springer 2022. p. 101–11. doi:10.1007/978-3-031-00868-9_6.

[146] Afzal I, Haq MZU, Ahmed S, Hirich A, Bazile D. Challenges and perspectives for integrating quinoa into the agri-food system. Plants. 2023;12(19):3361. doi:10.3390/PLANTS12193361. 37836099 PMC10574050

[147] Gomez-Pando LR, Aguilar-Castellanos E, Ibañez-Tremolada M. Quinoa (Chenopodium quinoa Willd.) Breeding. Adv Plant Breed Strategies:. 2019;5:259–316. doi:10.1007/978-3-030-23108-8_7.

[148] Haros CM, Muñoz L, Ortolá MD, Bazile D. Global trends in the worldwide expansion of quinoa cultivation. Biol Life Sci Forum. 2023;25(1):13. doi:10.3390/BLSF2023025013.

[149] Jacobsen SE. The situation for quinoa and its production in southern bolivia: from economic success to environmental disaster. Journal of Agronomy and Crop Science. 2011;197(5):390–99. doi:10.1111/J.1439-037X.2011.00475.X.

[150] Fikre A, Desmae H, Ahmed S. Tapping the economic potential of chickpea in Sub-Saharan Africa. Agronomy. 2020;10(11):1707. doi:10.3390/AGRONOMY10111707.

[151] Roorkiwal M, Bharadwaj C, Barmukh R, Dixit GP, Thudi M, Gaur PM, Chaturvedi SK, Fikre A, Hamwieh A, Kumar S, et al. Integrating genomics for chickpea improvement: achievements and opportunities. Theoretical and Applied Genetics. 2020;133(5):1703–20. doi:10.1007/s00122-020-03584-2. 32253478 PMC7214385

[152] Maphosa L, Richards MF, Norton SL, Nguyen GN. Breeding for abiotic stress adaptation in chickpea (Cicer arietinum L.): a comprehensive review. Crop Breed Genet Genomics. 2020. doi:10.20900/CBGG20200015.

[153] Ferede S, Girma N, Aliy S. Validating product concepts for optimizing chickpea breeding. Ethiopian Institute of Agricultural Research. 2019:09–10. http://www.eiar.gov.et.

[154] Singh M, Malhotra N, Singh K. Broadening the genetic base of cultivated chickpea following introgression of wild Cicer species-progress, constraints and prospects. Genet Resour Crop Evol. 2021;68(6):2181–205. doi:10.1007/s10722-021-01173-w.

[155] Verkaart S, Mausch K, Claessens L, Giller KE. A recipe for success? Learning from the rapid adoption of improved chickpea varieties in Ethiopia. International Journal of Agricultural Sustainability. 2019;17(1):34–48. doi:10.1080/14735903.2018.1559007. 30828358 PMC6382285

[156] Manjunatha L, Puyam A, Prema GU, Sanjay Bandi M, Kumar R, Keerthi MC, Dixit GP, Kavitha TR. Chickpea biotic stresses. Genomic Designing for Biotic Stress Resistant Pulse Crops. 2022:117–59. doi:10.1007/978-3-030-91043-3_2.

[157] Patil ND, Bains A, Sridhar K, Bhaswant M, Kaur S, Tripathi M, Lanterbecq D, Chawla P, Sharma M. Extraction, modification, biofunctionality, and food applications of chickpea (cicer arietinum) protein: an up-to-date review. Foods. 2024;13(9):1398. doi:10.3390/FOODS13091398. 38731769 PMC11083271

[158] Phiri CK, Njira K, Chitedze G. An insight of chickpea production potential, utilization and their challenges among smallholder farmers in Malawi – a review. Journal of Agriculture and Food Research. 2023;14:100713. doi:10.1016/J.JAFR.2023.100713.

[159] Mateva KI, Tan XL, Halimi RA, Chai HH, Makonya GM, Gao X, Shayanowako AIT, Ho WK, Tanzi AS, Farrant J, et al. Bambara groundnut (Vigna subterranea (L.) Verdc.). Neglected and Underutilized Crops: Future Smart Food. 2023:557–615. doi:10.1016/B978-0-323-90537-4.00021-1.

[160] Tan XL, Azam-Ali S, Von Goh E, Mustafa M, Chai HH, Ho WK, Mayes S, Mabhaudhi T, Azam-Ali S, Massawe F. Bambara groundnut: an underutilized leguminous crop for global food security and nutrition. Frontiers in Nutrition. 2020;7:601496. doi:10.3389/fnut.2020.601496. 33363196 PMC7758284

[161] Khan MMH, Rafii MY, Ramlee SI, Jusoh M, Al-Mamun M. Bambara Groundnut (Vigna subterranea L. Verdc): a crop for the new millennium, its genetic diversity, and improvements to mitigate future food and nutritional challenges. Sustainability. 2021b;13(10):5530. doi:10.3390/SU13105530.

[162] Gao X. Development of structured populations and breeding lines for trait analysis and improved varieties in Bambara Groundnut (Vigna subterranea L. Verdc). 2021.

[163] Olayide OE, Donkoh SA, Ansah IGK, Adzawla W, O’Reilly PJ, Mayes S, Feldman A, Halimi RA, Nyarko G, Ilori CO, et al. Assessing socioeconomic factors influencing production and commercialization of bambara groundnut as an indigenous climate resilient crop in nigeria. Handbook of Climate Change Resilience. 2018:1–19. doi:10.1007/978-3-319-71025-9_158-1.

[164] Amelework AB, Bairu MW. Advances in genetic analysis and breeding of cassava (Manihot esculenta Crantz): a Review. Plants. 2022;11(12):1617. doi:10.3390/PLANTS11121617. 35736768 PMC9228751

[165] Prempeh RNA, Amankwaah VA, Oppong A, Quain MD, Prempeh RNA, Amankwaah VA, Oppong A, Quain MD. Breeding cassava for end-user needs. Cassava - Recent Updates on Food, Feed, and Industry. 2023. doi:10.5772/INTECHOPEN.110363.

[166] Parmar A, Sturm B, Hensel O. Crops that feed the world: production and improvement of cassava for food, feed, and industrial uses. Food Security. 2017;9(5):907–27. doi:10.1007/s12571-017-0717-8.

[167] Thiele G, Dufour D, Vernier P, Mwanga ROM, Parker ML, Schulte Geldermann E, Teeken B, Wossen T, Gotor E, Kikulwe E, et al. A review of varietal change in roots, tubers and bananas: consumer preferences and other drivers of adoption and implications for breeding. International Journal of Food Science and Technology. 2021;56(3):1076–92. doi:10.1111/IJFS.14684. 33776222 PMC7983933

[168] Fu J, Hong Z, Huang W. Harnessing genomic tools for cassava improvement: advances and prospects. Biol Evidence. 2024;14:32–38. doi:10.5376/BE.2024.14.0005.

[169] Shioya A. Cassava commercialization and reactions in producing areas: a case study in Rural Eastern Cameroon. African Study Monographs Supplementary Issue. 2023;61:139–64. doi:10.14989/282793.

[170] Adebayo WG, Silberberger M. Poverty reduction, sustainable agricultural development, and the cassava value chain in Nigeria. The Palgrave Handbook of Agricultural and Rural Development in Africa. 2020:525–51. doi:10.1007/978-3-030-41513-6_24.

[171] Lyons JB, Bredeson JV, Mansfeld BN, Bauchet GJ, Berry J, Boyher A, Mueller LA, Rokhsar DS, Bart RS. Current status and impending progress for cassava structural genomics. Plant Mol Biol. 2022;109(3):177–91. doi:10.1007/s11103-020-01104-w. 33604743 PMC9162999

[172] Bowser TJ, Ojwang F, Sahs R, Brandenberger L. Promotion of Orange flesh sweet potato by demonstration of acceptance and food product development. Afr J Food Sci 2017;11(12):383–88. doi:10.5897/AJFS2017.1647.

[173] Mourtala IZM, Innocent NM, Habibou M, Oselebe H. Recent progress in breeding for beta-carotene, dry matter content and sugar in sweet potato [Ipomoea Batatas (L.) Lam]-A Review. European Journal of Agriculture and Food Sciences. 2023;5(1):6–13. doi:10.24018/EJFOOD.2023.5.1.551.

[174] Adekambi SA, Okello JJ, Low J, Abidin PE, Carey E. Does vitamin A rich Orange-fleshed sweetpotato adoption improve household level diet diversity? Evidence from Ghana and Nigeria. African Journal of Science, Technology, Innovation and Development. 2023;15(1):59–68. doi:10.1080/20421338.2021.2015172.

[175] Sajeev MS, Padmaja G, Sheriff JT, Jyothi AN. Entrepreneurial opportunities in tuber crops processing. Entrep Skill Dev Hortic Process. 2021:295–322. doi:10.1201/9781003246138-14/ENTREPRENEURIAL-OPPORTUNITIES-TUBER-CROPS-PROCESSING-SAJEEV-PADMAJA-SHERIFF-JYOTHI.

[176] Girard AW, Brouwer A, Faerber E, Grant FK, Low JW. Orange-fleshed sweetpotato: strategies and lessons learned for achieving food security and health at scale in Sub-Saharan Africa. Open Agriculture. 2021;6(1):511–36. doi:10.1515/opag-2021-0034.

[177] Yan M, Nie H, Wang Y, Wang X, Jarret R, Zhao J, Wang H, Yang J. Exploring and exploiting genetics and genomics for sweetpotato improvement: status and perspectives. Plant Communications. 2022;3(5):100332. doi:10.1016/J.XPLC.2022.100332. 35643086 PMC9482988

[178] Mwanga ROM, Andrade MI, Carey EE, Low JW, Craig Yencho G, Grüneberg WJ. Sweetpotato (Ipomoea batatas L.). Genetic Improvement of Tropical Crops. 2017:181–218. doi:10.1007/978-3-319-59819-2_6.

[179] Da Silva Pereira G, Gemenet DC, Mollinari M, Olukolu BA, Wood JC, Diaz F, Mosquera V, Gruneberg WJ, Khan A, Buell CR, et al. Multiple QTL mapping in autopolyploids: a random-effect model approach with application in a hexaploid sweetpotato full-sib population. Genetics. 2020;215(3):579–95. doi:10.1534/GENETICS.120.303080. 32371382 PMC7337090

[180] Oloka BM, da Silva Pereira G, Amankwaah VA, Mollinari M, Pecota KV, Yada B, Olukolu BA, Zeng ZB, Craig Yencho G. Discovery of a major QTL for root-knot nematode (Meloidogyne incognita) resistance in cultivated sweetpotato (Ipomoea batatas). TAG. Theoretical and Applied Genetics. Theoretische Und Angewandte Genetik. 2021;134(7):1945–55. doi:10.1007/S00122-021-03797-Z. 33813604 PMC8263542

[181] Haque E, Tabuchi H, Monden Y, Suematsu K, Shirasawa K, Isobe S, Tanaka M. QTL analysis and GWAS of agronomic traits in sweetpotato (Ipomoea batatas L.) using genome wide SNPs. Breed Sci. 2020;70(3):283–91. doi:10.1270/JSBBS.19099. 32714050 PMC7372034

[182] Nie H, Park H, Kim S, Kim D, Kim S, Kwon SY, Kim SH. Genetic diversity assessment and genome-wide association study reveal candidate genes associated with component traits in sweet potato (Ipomoea batatas (L.) Lam). Molecular Genetics and Genomics. 2023;298(3):653–67. doi:10.1007/s00438-023-02007-3. 36943475

[183] Wang H, Wu Y, Zhang Y, Yang J, Fan W, Zhang H, Zhao S, Yuan L, Zhang P. CRISPR/Cas9-based mutagenesis of starch biosynthetic genes in sweet potato (Ipomoea Batatas) for the improvement of starch quality. Int J Mol Sci. 2019;20(19):4702. doi:10.3390/IJMS20194702. 31547486 PMC6801948

[184] Gemenet DC, Lindqvist-Kreuze H, De Boeck B, da Silva Pereira G, Mollinari M, Zeng ZB, Craig Yencho G, Campos H. Sequencing depth and genotype quality: accuracy and breeding operation considerations for genomic selection applications in autopolyploid crops. Theoretical and Applied Genetics. 2020;133(12):3345–63. doi:10.1007/s00122-020-03673-2. 32876753 PMC7567692

[185] Xie F, Burklew CE, Yang Y, Liu M, Xiao P, Zhang B, Qiu D. De novo sequencing and a comprehensive analysis of purple sweet potato (Impomoea batatas L.) transcriptome. Planta. 2012;236(1):101–13. doi:10.1007/s00425-012-1591-4. 22270559

[186] Mabhaudhi T, Chimonyo VGP, Hlahla S, Massawe F, Mayes S, Nhamo L, Modi AT. Prospects of orphan crops in climate change. Planta. 2019;250(3):695–708. doi:10.1007/s00425-019-03129-y. 30868238 PMC6667417

[187] Borelli T, Rana J, Gauchan D, Mendonce S, Hunter D. Inclusion criteria for underutilized food plants in nutrition-sensitive programming. Neglected Plant Foods South Asia Exploring Valorizing Nature Feed Hunger. 2023:73–100. doi:10.1007/978-3-031-37077-9_4.

[188] Mabhaudhi T, Hlahla S, Chimonyo VGP, Henriksson R, Chibarabada TP, Murugani VG, Groner VP, Tadele Z, Sobratee N, Slotow R, et al. Diversity and diversification: ecosystem services derived from underutilized crops and their co-benefits for sustainable agricultural landscapes and resilient food systems in Africa. Frontiers in Agronomy. 2022;4:859223. doi:10.3389/fagro.2022.859223. 37680880 PMC7615041

[189] Knez M, Ranic M, Gurinovic M, Glibetic M, Savic J, Mattas K, Yercan M. Causes and conditions for reduced cultivation and consumption of underutilized crops: is there a solution? Sustainability. 2023;15(4):3076. doi:10.3390/SU15043076.

[190] Chongtham SK, Devi EL, SamantaraYasin KJK, Wani SH, Mukherjee S, Razzaq A, Bhupenchandra I, Jat AL, Singh LK, Kumar A. Orphan legumes: harnessing their potential for food, nutritional and health security through genetic approaches. Planta. 2022;256(2):1–28. doi:10.1007/S00425-022-03923-1. 35767119

[191] Leakey RRB, Tientcheu Avana ML, Awazi NP, Assogbadjo AE, Mabhaudhi T, Hendre PS, Degrande A, Hlahla S, Manda L. The future of food: domestication and commercialization of indigenous food crops in Africa over the third decade (2012–2021). Sustainability (Switzerland). 2022;14(4):2355. doi:10.3390/SU14042355/S1.

[192] Onomu AR. Pitfalls and potential pathways to commercialization of indigenous food crops, fruits, and vegetables in Africa. Asian Journal of Agriculture and Rural Development. 2023;13(1):25–38. doi:10.55493/5005.v13i1.4716.

